# Exploring the Substrate Flexibility of GrsB Thioesterase Leads to the Structural Reassignment of a Gramicidin S Variant

**DOI:** 10.1002/cbic.202500412

**Published:** 2025-08-26

**Authors:** Sho Konno, Tomoe Mizuguchi, Atsuko Suzuki, Miyu Tanaka, Fumihiro Ishikawa, Akihiro Taguchi, Atsuhiko Taniguchi, Genzoh Tanabe, Yoshio Hayashi

**Affiliations:** ^1^ School of Pharmacy Tokyo University of Pharmacy and Life Sciences 1432–1 Horinouchi Hachioji Tokyo 192‐0392 Japan; ^2^ Faculty of Pharmacy Kindai University 3‐4‐1 Kowakae Higashi‐osaka Osaka 577–8502 Japan; ^3^ School of Life Sciences Tokyo University of Pharmacy and Life Sciences 1432–1 Horinouchi Hachioji Tokyo 192‐0392 Japan

**Keywords:** enzymatic cyclization, gramicidin S, nonribosomal peptide synthetase, stereochemical reassignment, thioesterase domain

## Abstract

Gramicidin S (GS) is a cyclic decapeptide derived from two pentapeptides. The C‐terminal thioesterase (TE) domain of gramicidin S synthetase B (GrsB) dimerizes precursor pentapeptides and cyclizes the resulting linear decapeptide. Recently, a GS variant (GS‐SA), in which a single D‐Phe is replaced by L‐Ser(Allyl), is reported via precursor‐directed biosynthesis in a native GS producer. To understand how GrsB‐TE processes such modified precursors, its substrate specificity using synthetic linear peptides is investigated. GrsB‐TE cyclizes a substrate containing L‐Ser(Allyl) at position 6 but not at position 1. However, the enzymatically synthesized GS‐SA shows a different high‐performance liquid chromatography retention time than that of the reported GS variant. Further structural and functional analyses, including ^1^H nuclear magnetic resonance, antimicrobial assays, and circular dichroism spectroscopy, reveal that the reported GS‐SA contained D‐Ser(Allyl) rather than L‐Ser(Allyl). These findings reveal a previously unrecognized stereochemical flexibility in GrsB‐TE and support the structural revision of the reported GS variant.

## Graphical abstract

Investigation of the substrate specificity of gramicidin S synthetase B‐thioesterase reveals stereochemical flexibility, enabling the production of gramicidin S (GS) analogs with L‐ or D‐Ser(Allyl). Structural reassignment of a GS variant (GS‐SA) obtained from precursor‐directed biosynthesis is achieved using liquid chromatography‐mass spectrometry, ^1^H nuclear magnetic resonance, and antimicrobial assays.

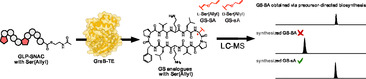

## 1 Introduction

Nonribosomal peptides (NRPs) represent a structurally diverse class of natural products with significant pharmacological potential.^[^ ^1^ ^,^ ^2^ ^]^ These bioactive peptides often exhibit complex architectures, including macrocyclic structures, nonproteogenic amino acids, and various chemical modifications that enhance their biological activity. Macrocyclic structures confer improved stability, membrane permeability, and target specificity, making them attractive scaffolds for drug development.^[^ ^3^ ^,^ ^4^ ^]^ This remarkable structural diversity is orchestrated by nonribosomal peptide synthetases (NRPSs), which are large, multimodular enzymes that utilize an assembly line, incorporating diverse amino acid building blocks, and elongating peptide chains.^[^ ^5^ ^,^ ^6^ ^]^

**Figure 1:**
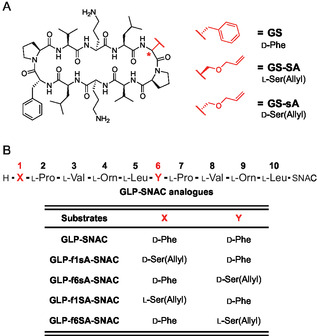
A) Structure of gramicidin S (GS) and its analogs. GS‐SA and GS‐sA represent GS variants containing L‐Ser(Allyl) (SA) and D‐Ser(Allyl) (sA), respectively. B) Structure of the gramicidin S‐linear peptide *N*‐acetylcysteamine thioester (GLP‐SNAC) and its analogs. Lowercase letters indicate D‐amino acids.

Gramicidin S (GS) is one of the oldest antibiotics isolated from *Bacillus brevis* in the 1940s.^[^ ^7^ ^,^ ^8^ ^]^ The cyclic decapeptide, composed of two identical pentapeptide units, cyclo(D‐Phe‐Pro‐Val‐Orn‐Leu)_2_, formed a symmetrical amphipathic structure (**Figure** **1**A). This dimeric structure adopts a *β*‐sheet‐like conformation stabilized by four intramolecular hydrogen bonds, which play a crucial role in its strong antimicrobial activity, derived from cytoplasmic membrane disruption.^[^ ^9^ ^,^ ^10^ ^]^ While the clinical use of GS is limited owing to its hemolytic activity, its mode of action of GS may explain why no resistance has been observed in over 70 years.^[^ ^11^ ^]^ Given its potent bioactivity and well‐defined structural template, GS has served as a valuable scaffold for antibiotic development, and numerous structure–activity relationship studies have been conducted to separate antimicrobial activity from hemolytic side effects.^[^ ^12^ ^,^ ^13^ ^]^

These unique structural features are biosynthesized by two NRPSs, GrsA and GrsB (Figure S1, Supporting Information).^[^ ^14^ ^,^ ^15^ ^]^ GrsA is a single‐module enzyme comprising an adenylation (A) domain, a peptidyl carrier protein (PCP) domain, and an epimerase (E) domain. The GrsA‐A domain selects and activates L‐Phe to form L‐Phe‐AMP, which is then transferred to the phosphopantetheine arm of the PCP domain. The tethered L‐Phe is then epimerized to D‐Phe by the E domain. This D‐Phe serves as the starting unit for chain elongation and is specifically recognized and coupled with L‐Pro by the condensation (C) domain in module 2 of GrsB.^[^ ^16^ ^]^ In the final step of peptide assembly, the thioesterase (TE) domain at the *C*‐terminus of GrsB dimerizes with two pentapeptides and cyclizes the resulting linear decapeptide to yield GS. NRPS‐TEs with broad substrate tolerance are promising biocatalysts for generating natural product‐like cyclic peptide libraries.^[^ ^17^, ^18^, ^19^ ^–^ ^20^ ^]^ Previous systematic characterization of GrsB‐TE revealed that recombinant GrsB‐TE can catalyze head‐to‐tail cyclization and produce 6–15 mer cyclic peptides from the corresponding synthetic linear peptide substrates.^[^ ^21^ ^,^ ^22^ ^]^ It also accepts synthetic substrates bearing side‐chain substitutions, demonstrating broad tolerance toward both ring size and side‐chain functionalities.^[^ ^22^ ^,^ ^23^ ^]^ Thus, characterization of GrsB‐TE provides insights into TE substrate specificity, and further investigation of its stereochemical and structural tolerance will facilitate the understanding of NRPS cyclization.

Recently, new GS variants were produced via precursor‐directed biosynthesis (PDB).^[^ ^24^ ^]^ By leveraging the ability of the GrsA‐A domain to recognize L‐Ser(Allyl) as a surrogate substrate, feeding L‐Ser(Allyl) into cultures of *Aneurinibacillus migulanus* ATCC 9999 resulted in the incorporation of a single L‐Ser(Allyl) residue into the GS structure, replacing D‐Phe and yielding the novel compound GS‐L‐Ser(Allyl) (GS‐SA, Figure 1A). L‐Amino acid was incorporated at this position despite the presence of the E‐domain, as previously observed with *O*‐propargyl‐L‐Tyr, which was predominantly incorporated without epimerization in the GrsA‐W239S/GrsB1 system.^[^ ^25^ ^]^ The discovery of GS‐SA led us to investigate the dimerization and cyclization of the GS variant. Here, we investigated the substrate specificity of GrsB‐TE toward mono D‐Phe‐substituted decapeptide analogs, focusing on its ability to cyclize these variants. Our in vitro enzymatic analysis using synthetic linear peptide substrates revealed that GrsB‐TE can produce both GS‐SA and GS‐D‐Ser(Allyl) (GS‐sA, Figure 1A) analogs. Unexpectedly, the high‐performance liquid chromatography (HPLC) retention time of the enzymatically synthesized GS‐SA did not match that of the reported GS‐SA obtained from PDB. Our comprehensive structural and functional analyses, including ^1^H nuclear magnetic resonance (NMR), antibacterial assays, and circular dichroism (CD) spectroscopy, confirmed that the reported GS‐SA contained D‐Ser(Allyl) instead of L‐Ser(Allyl), leading to a revision of its originally proposed structure.

## 2 Results and Discussion

**Figure 2:**
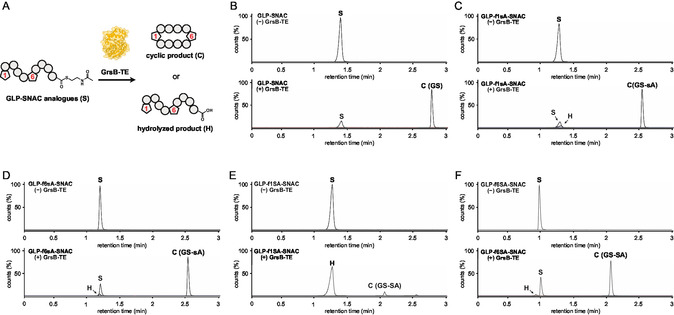
Enzymatic assay of GLP‐SNAC analogs with GrsB‐TE. A) The schematic illustration of the GrsB‐TE‐catalyzed reaction. Substrate (200 µM) was incubated with GrsB‐TE (10 µM) in 20 mM Tris (pH 7.0) at 24 °C for 2 h. The reactions were monitored by liquid chromatography‐mass spectrometry (LC‐MS) and assigned as substrate (S), cyclic product (C), and hydrolyzed product (H). B) LC‐MS traces of reactions containing GLP‐SNAC, without or with GrsB‐TE. Extracted ion chromatograms (EICs) are shown for S ([M + 2H]^2+^ *m/z* 630.8805), H ([M + 2H]^2+^ *m/z* 580.3656), and C ([M + 2H]^2+^ *m/z* 571.3603). C–F) LC‐MS traces of reactions containing GLP‐SNAC analogs, without or with GrsB‐TE. EICs were extracted for S ([M + 2H]^2+^ *m/z* 620.8780), H ([M + 2H]^2+^ *m/z* 570.3630), and C ([M + 2H]^2+^ *m/z* 561.3577). The *y*‐axis of each panel shows the relative intensity, with the combined peak areas of S, H, and C normalized to 100%. This normalization allows for direct visual comparison of product distributions.

**Figure 3:**
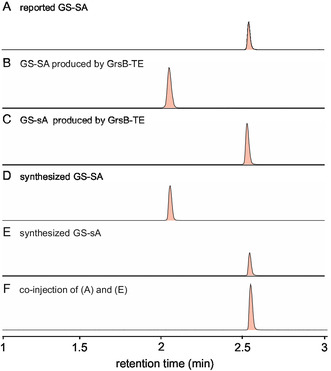
Comparison of retention times of GS‐Ser(Allyl) analogs. Extracted ion chromatograms were obtained at [M + 2H]^2+^ *m/z* 561.3577. A) Reported GS‐SA. B) Enzymatically synthesized GS‐SA. C) Enzymatically synthesized GS‐sA. D) Chemically synthesized GS‐SA. E) Chemically synthesized GS‐sA. F) Co‐injection of reported GS‐SA and chemically synthesized GS‐sA.

First, we investigated whether GrsB‐TE accepts synthetic substrates in which each of the two D‐Phe residues was individually replaced with either L‐Ser(Allyl) or D‐Ser(Allyl) (**Figure** **2**A). To achieve this, we synthesized gramicidin S linear peptide *N*‐acetylcysteamine thioester (GLP‐SNAC) and the corresponding analogs (Figure 1B and Schemes S1–S3, Supporting Information). The SNAC group served as the minimal scaffold recognized by the NRPS‐TE domains.^[^ ^26^ ^]^ Recombinant GrsB‐TE with an appropriate *N*‐terminal linker was cloned and expressed because an unusually long linker has been reported to be important for its stability and activity (Figure S2, Supporting Information).^[^ ^22^ ^]^ The summary of the in vitro enzymatic assays is shown in **Table** **1**. Incubation of GLP‐SNAC (200 µM) and GrsB‐TE (10 µM) for 2 h produced GS with a 77% yield (Figures 2B and S3, Supporting Information). The reactions of GLP‐f1sA‐SNAC and GLP‐f6sA‐SNAC containing D‐Ser(Allyl) with GrsB‐TE yielded GS‐sA in 73 and 72%, respectively, indicating that GrsB‐TE can catalyze the cyclization of both D‐Ser(Allyl)‐substituted substrates (Figures 2C,D and S4, S5, Supporting Information). Next, we tested whether GrsB‐TE cyclizes L‐Ser(Allyl)‐containing substrates. The incubation of GLP‐f1SA‐SNAC with GrsB‐TE resulted in a hydrolysis product (Figures 2E and S6, Supporting Information). In contrast, GS‐SA was efficiently produced in the reaction of GLP‐f6SA‐SNAC with GrsB‐TE (Figures 2F and S7, Supporting Information). These results support a previous report showing that the D configuration at position 1 is crucial for GrsB‐TE‐mediated macrocyclization.^[^ ^22^ ^]^ Additionally, our results demonstrated that GrsB‐TE efficiently cyclized the L‐Ser(Allyl)‐containing substrate when introduced at position 6. To verify whether the GS‐SA produced by GrsB‐TE corresponded to the reported GS‐SA derived from the PDB, we compared their retention times; however, the results were inconsistent. Notably, the retention time of the reported GS‐SA was identical to that of GS‐sA (**Figure** **3**A–C).

**Table 1:** Summary of the reactions with GLP‐SNAC analogs and GrsB‐TE.

Substrates	Cyclic product	Cyclic product [%]a)	Hydrolyzed product [%]a)
GLP‐SNAC	GS	77	>1
GLP‐f1sA‐SNAC	GS‐sA	73	7
GLP‐f6sA‐SNAC	GS‐sA	72	4
GLP‐f1SA‐SNAC	GS‐SA	3	96
GLP‐f6SA‐SNAC	GS‐SA	68	31

a) Yields (%) were calculated based on the [M + 2H]^2+^ peak area of each peptide measured at 2 h, using the following formula: [peak area of the desired product] / [sum of peak areas of the substrate, cyclic product, and hydrolyzed product] × 100. Calibration curves for GLP‐SNAC and GS (based on [M + 2H]^2+^) showed comparable slopes, supporting the use of relative MS peak areas to estimate the yields.

To investigate the structures of GS analogs, we chemically synthesized GS‐SA and GS‐sA (Scheme S4, Supporting Information). Starting with H‐L‐Leu‐O‐Trt(2‐Cl)‐resin, the protected linear decapeptides were synthesized using Fmoc‐solid phase peptide synthesis, followed by cleavage from the resin with 20% HFIP/CH_2_Cl_2_. Head‐to‐tail macrocyclization of the protected peptides was achieved using PyBOP/HOBt under highly diluted conditions (≈0.3 mM), as described in the literature.^[^ ^27^ ^]^ Global deprotection, followed by HPLC purification, afforded GS‐SA (78%) and GS‐sA (29%). GS‐SA and GS‐sA produced by GrsB‐TE showed HPLC retention times identical to those of the chemically synthesized standards (Figures 3B–E), demonstrating that the GrsB‐TE‐mediated products are the desired head‐to‐tail cyclic products. Notably, co‐injection of the reported GS‐SA with the chemically synthesized GS‐sA resulted in a single peak (Figure 3F). Furthermore, a comparison of the ^1^H NMR spectra of the reported GS‐SA with those of chemically synthesized GS‐SA and GS‐sA revealed that the spectrum of GS‐sA closely matched that of the reported GS‐SA (Figure S8, Supporting Information). These data suggested that the reported GS‐SA variant was GS‐sA.

Next, we conducted an antimicrobial assay of GS analogs against *Bacillus subtilis* ATCC 6051. GS was synthesized via the same route as that for the GS analogs (Scheme S4, Supporting Information). As shown in **Table** **2** and Figure S9 (Supporting Information), GS exhibited a minimal inhibitory concentration (MIC) of 0.5 µg mL^−1^, which was slightly lower than the previously reported value but remained within the range of experimental variation. The MIC value of GS‐sA was 1.0 µg mL^−1^, consistent with the previously observed trend that the reported GS‐SA is slightly less potent than GS.^[^ ^24^ ^]^ In contrast, GS‐SA showed a markedly decreased antimicrobial activity (MIC = 64 µg mL^−1^), indicating that the reverse configuration at this position significantly impairs biological activity. Although the stereochemical assignment of the Ser(Allyl) residue in the reported GS‐SA was conducted using advanced Marfey's method, the extracted ion chromatogram of the corresponding derivative was notably weak, and no co‐injection was performed. Given that retention times can vary depending on experimental conditions, and that acid hydrolysis may lead to degradation of the *O*‐Allyl moiety,^[^ ^28^ ^]^ resulting in reduced signal intensity of the Ser(Allyl) derivative, the possibility of misassignment cannot be excluded. Based on direct comparison of chemically synthesized L‐ and D‐Ser(Allyl) analogs with the reported GS‐SA by ^1^H NMR and co‐injection in HPLC, along with the antimicrobial assay results, we conclude that the reported GS‐SA corresponds to the D‐configured analog, GS‐sA.

**Table 2:** Antimicrobial activities of GS, GS‐SA, and GS‐sA.

Compoundsa)	MICs [µg mL^−1^]
GS	0.5 (1.0)b)
GS‐SA	64
GS‐sA	1.0
reportedGS‐SA	(4.0)b)

a) MIC values against *Bacillus subtilis* ATCC 6051 were determined using microplate dilution method;

b) MIC values reported in literature.^[^ ^24^ ^]^

**Figure 4:**
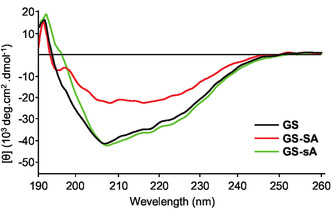
CD spectra of GS (black), GS‐SA (red), and GS‐sA (green) in MeOH.

As mentioned above, the *β*‐sheet conformation of GS plays a crucial role in its antibacterial activity. We speculated that the incorporation of L‐Ser(Allyl) at the D‐Phe position might influence this specific secondary structure, potentially altering the conformation required for antimicrobial activity. To evaluate this possibility, we conducted circular dichroism (CD) spectroscopy to compare the secondary structures of GS and its analogs. The CD spectrum of GS exhibited two negative peaks at 206 and 220 nm, consistent with the CD spectrum of GS reported previously (**Figure** **4**).^[^ ^29^ ^]^ CD spectroscopy revealed that GS‐sA exhibited a spectrum nearly identical to that of GS, suggesting that GS‐sA retained its overall secondary structure (Figure 4). In contrast, GS‐SA displayed a distinct CD spectrum (Figure 4), indicating a significant disruption in its *β*‐sheet conformation. This structural change is further supported by ^1^H NMR analysis in CD_3_OD, which revealed the absence of amide proton signals typically observed in GS (Figure S8, Supporting Information). Since these signals arise from intramolecular hydrogen bonds that stabilize the *β*‐sheet conformation, their disappearance indicates a disruption of the *β*‐sheet structure in GS‐SA. The *β*‐sheet conformation in GS contributes to its amphiphilic nature, which plays an important role in membrane interaction and antimicrobial activity.^[^ ^13^ ^]^ The structural change in GS‐SA likely reduces this amphiphilicity, providing a possible explanation for the loss of antibacterial activity.

## 3 Conclusion

In this study, we investigated the substrate specificity of GrsB‐TE using synthetic linear substrates in which a single D‐Phe residue was replaced by L‐ or D‐Ser(Allyl). GrsB‐TE efficiently catalyzed the cyclization of D‐Ser(Allyl)‐containing substrates substituted at either position 1 or 6, indicating that both positions are tolerated. In contrast, L‐Ser(Allyl)‐containing substrates were cyclized only when substitution occurred at position 6. Next, we compared the enzymatically synthesized products with the reported GS‐SA variant obtained using PDB. A comparison of HPLC retention times, ^1^H NMR, and antimicrobial assays revealed that the previously reported GS‐SA variant contained D‐Ser(Allyl) and not L‐Ser(Allyl). Moreover, we demonstrated that the incorporation of L‐Ser(Allyl) at the D‐Phe position disrupted the *β*‐sheet conformation, leading to a loss of antimicrobial activity. This work not only revises the structure of the reported GS variant but also expands the known substrate tolerance of GrsB‐TE, highlighting its potential as a biocatalyst for the chemoenzymatic synthesis of novel GS analogs.

## Supporting Information

The authors have cited additional references within the Supporting Information.^[30–31]^

## Conflict of Interest

The authors declare no conflict of interest.

## Supporting information

Supplementary Material

## Data Availability

The data that support the findings of this study are available in the supplementary material of this article.
