# Supplementary material for: Exploring the Substrate Flexibility of GrsB Thioesterase Leads to the Structural Reassignment of a Gramicidin S Variant
Source: Chembiochem. 2025 Aug 26;26(22):e202500412. doi: 10.1002/cbic.202500412 (PMC12630999; doi:10.1002/cbic.202500412)

Supporting Information

Exploring the Substrate Flexibility of GrsB Thioesterase Leads to the Structural Reassignment of a Gramicidin S Variant

Sho Konno,[1,#,*] Tomoe Mizuguchi,[1,#] Atsuko Suzuki,[1] Miyu Tanaka,[1] Fumihiro Ishikawa,[2] Akihiro Taguchi,[1] Atsuhiko Taniguchi,[1] Genzoh Tanabe[2] and Yoshio Hayashi[1,3,*]

1. School of Pharmacy, Tokyo University of Pharmacy and Life Sciences, Hachioji, Tokyo 192-0392, Japan.
2. Faculty of Pharmacy, Kindai University, Higashi-osaka, Osaka 577-8502, Japan
3. School of Life Sciences, Tokyo University of Pharmacy and Life Sciences, Hachioji, Tokyo 192-0392, Japan.

E-mail: skonno@toyaku.ac.jp, yhayashi@toyaku.ac.jp

* Corresponding authors
# Equal contributors

Contents: **Page**

Supporting Figure S1	S3
Supporting Figure S2	S4
Supporting Figure S3	S5
Supporting Figure S4	S6
Supporting Figure S5	S7
Supporting Figure S6	S8
Supporting Figure S7	S9
Supporting Figure S8	S10
Supporting Figure S9	S11
Biochemical procedures	S12-S13
Chemical synthetic procedures	S14-S-25
Scheme S1	S14
Scheme S2	S16
Scheme S3	S17
Scheme S4	S18
HPLC chromatograms showing the purity	S26-S29
^{1}H NMR and ^{13}C NMR spectra	S30-S35

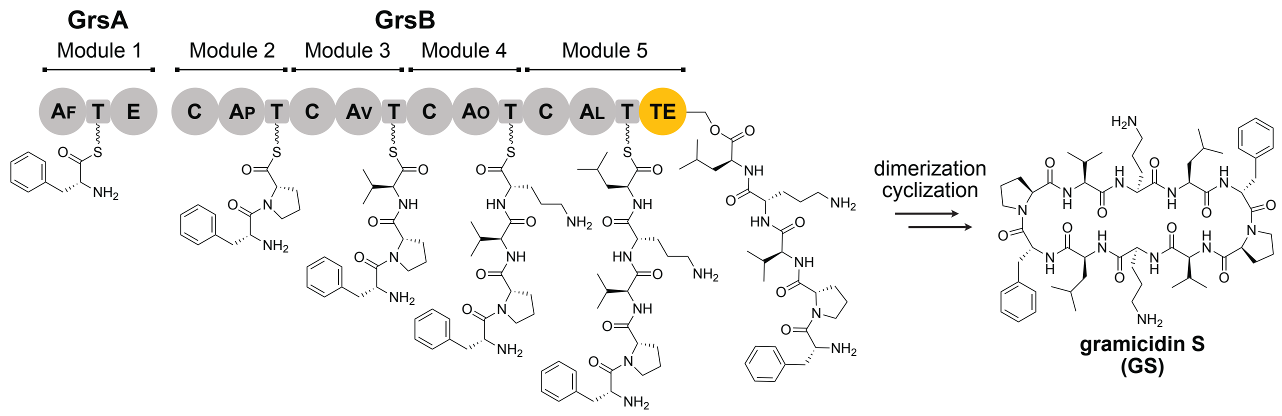

Figure S1. Biosynthesis of gramicidin S. Modules are comprised thiolation (T), adenylation (A_F: L-Phe; A_P: L-Pro; A_V: L-Val; A_O: L-Orn; A_L: L-Leu specific adenylating domains), epimerization (E), condensation (C), and thioesterase (TE) domains. GrsB-TE domain catalyzes the dimerization of two pentapeptides, followed by head-to-tail cyclization to yield gramicidin S.

MGSSHHHHHHSSGLVPRGSHMVNVEADREALSLNGEKQRKNIELPILLNEETDRNVFLFAPIGA QGVFYKKLAEQIPTASLYGFDFIEDDDRIQQYIESMIQTQSDGQYVLIGYSSGGNLAFEVAKEME RQGYSVSDLVLFDVYWKGKVFEQTKEEEEENIKIIMEELRENPGMFNMTREDFELYFANEFVKQ SFTRKMRKYMSFYTQLVNYGEVEATIHLIQAEFEEEKIDENEKADEEEKTYLEEKWNEKAWNKA AKRFVKYNGYGAHSNMLGGDGLERNSSILKQILQGTFVVK

Figure S2. Amino acid sequence of recombinant GrsB-TE used in this study. The residues in bold originate from pET28a vector. The sequence in red indicates the start of GrsB-TE.

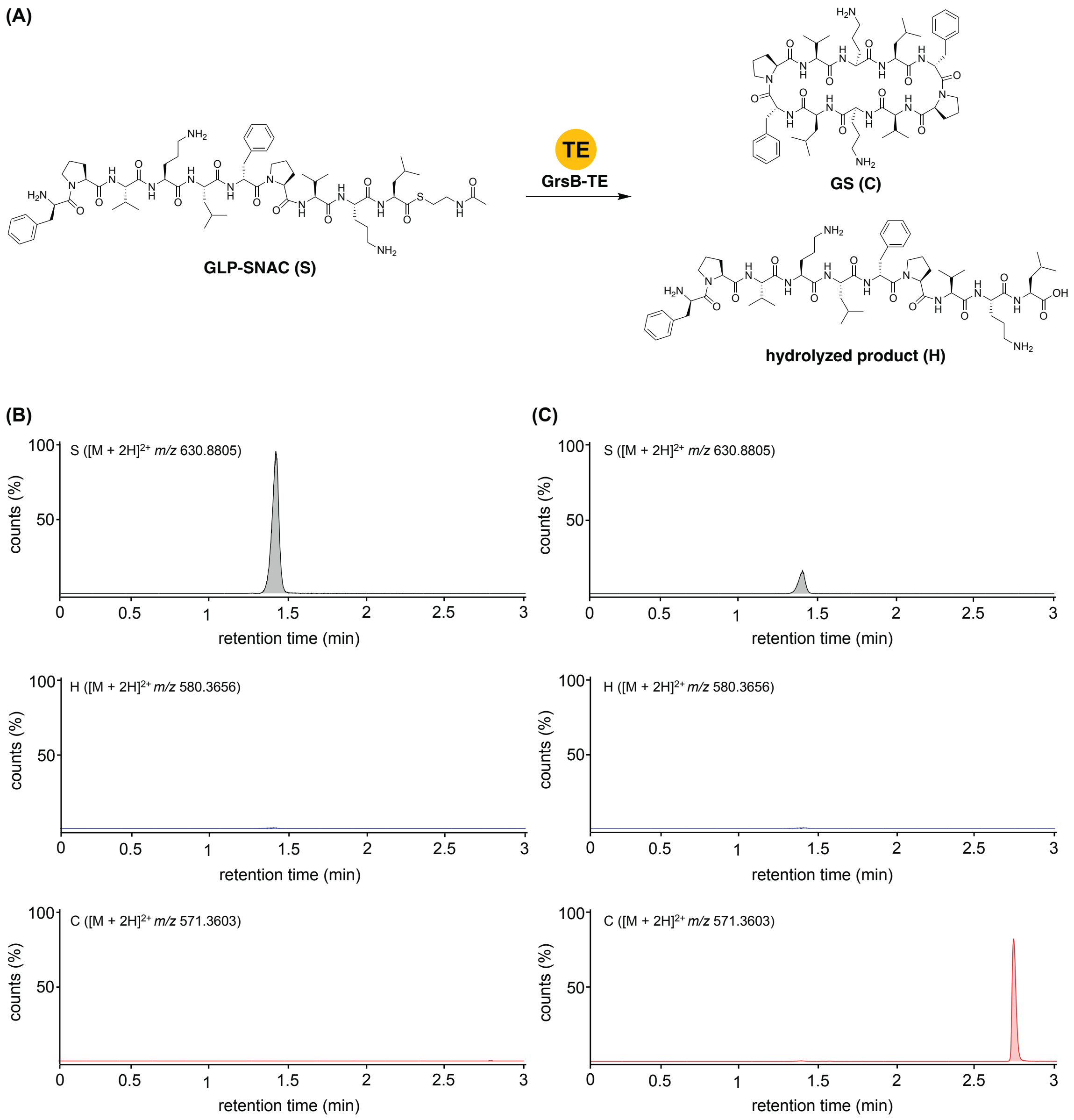

Figure S3. (A) *In vitro* enzymatic assay with GLP-SNAC. Separated LC/MS traces of the reactions containing GLP-SNAC, without **(B)** or with **(C)** GrsB-TE, corresponding to Figure 2B. The top, middle, and bottom panels show EICs of GLP-SNAC (S, $[M + 2H]^{2+}$ *m/z* 630.8805), the hydrolyzed product (H, $[M + 2H]^{2+}$ *m/z* 580.3656), and GS (C, $[M + 2H]^{2+}$ *m/z* 571.3603), respectively.

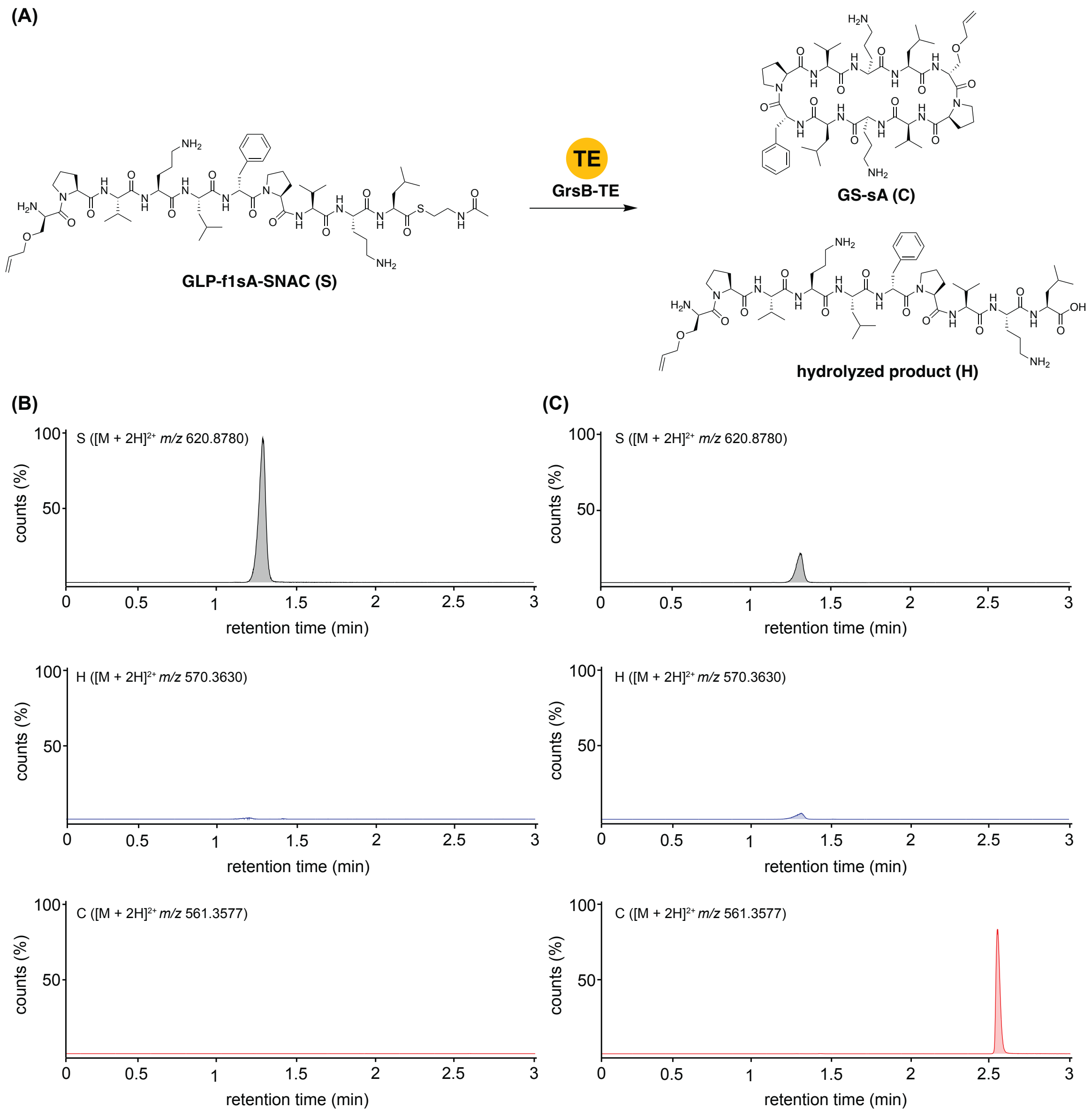

Figure S4. (A) *In vitro* enzymatic assay with GLP-f1sA-SNAC. Separated LC/MS traces of the reactions containing GLP-f1sA-SNAC, without **(B)** or with **(C)** GrsB-TE, corresponding to Figure 2C. The top, middle, and bottom panels show EICs of GLP-f1sA-SNAC (S, [M + 2H]$^{2+}$ *m/z* 620.8780), the hydrolyzed product (H, [M + 2H]$^{2+}$ *m/z* 570.3630), and GS-sA (C, [M + 2H]$^{2+}$ *m/z* 561.3577), respectively.

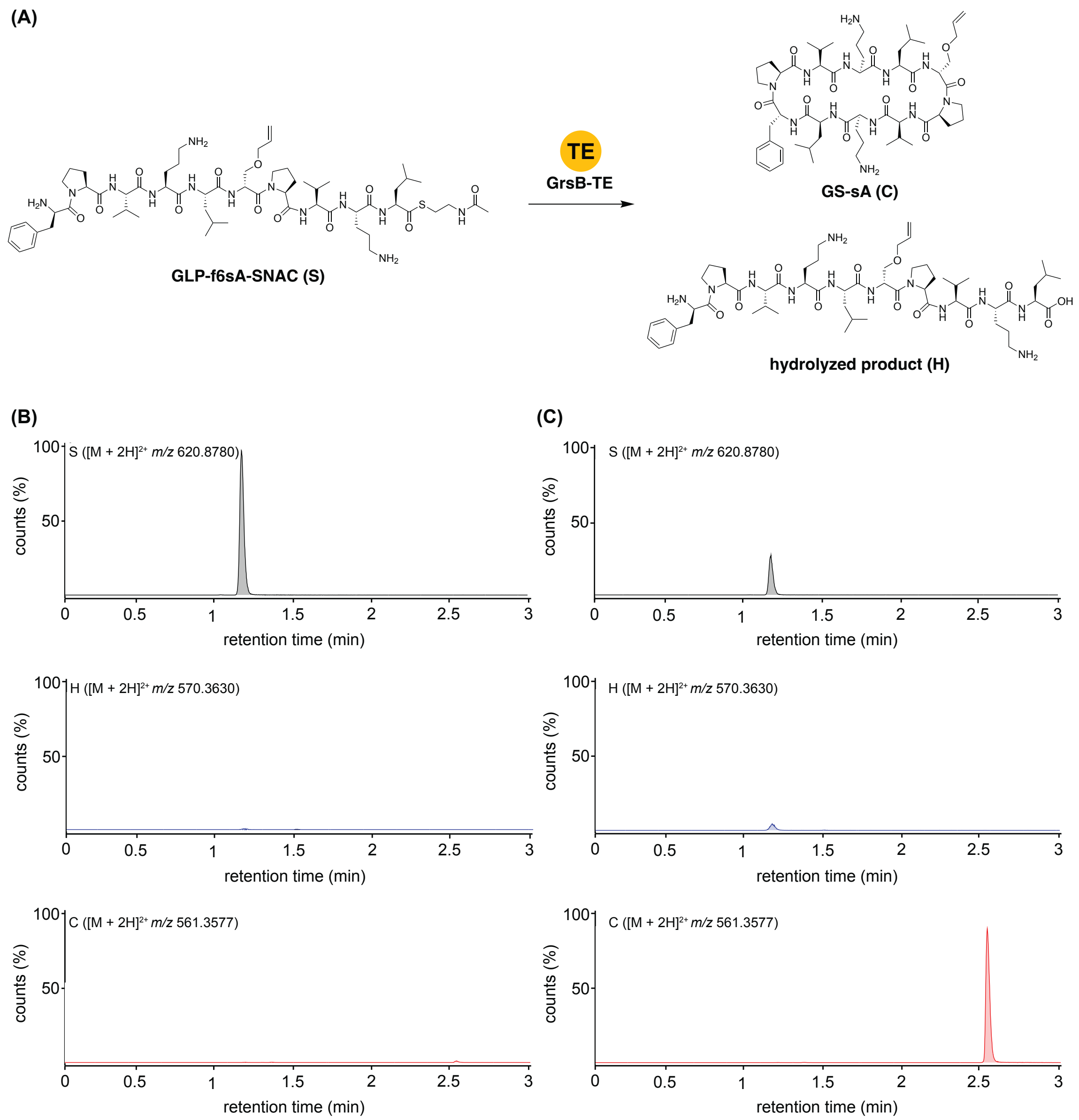

Figure S5. (A) *In vitro* enzymatic assay with GLP-f6sA-SNAC. Separated LC/MS traces of the reactions containing GLP-f6sA-SNAC, without **(B)** or with **(C)** GrsB-TE, corresponding to Figure 2D. The top, middle, and bottom panels show EICs of GLP-f6sA-SNAC (S, $[M + 2H]^{2+}$ *m/z* 620.8780), the hydrolyzed product (H, $[M + 2H]^{2+}$ *m/z* 570.3630), and GS-sA (C, $[M + 2H]^{2+}$ *m/z* 561.3577), respectively.

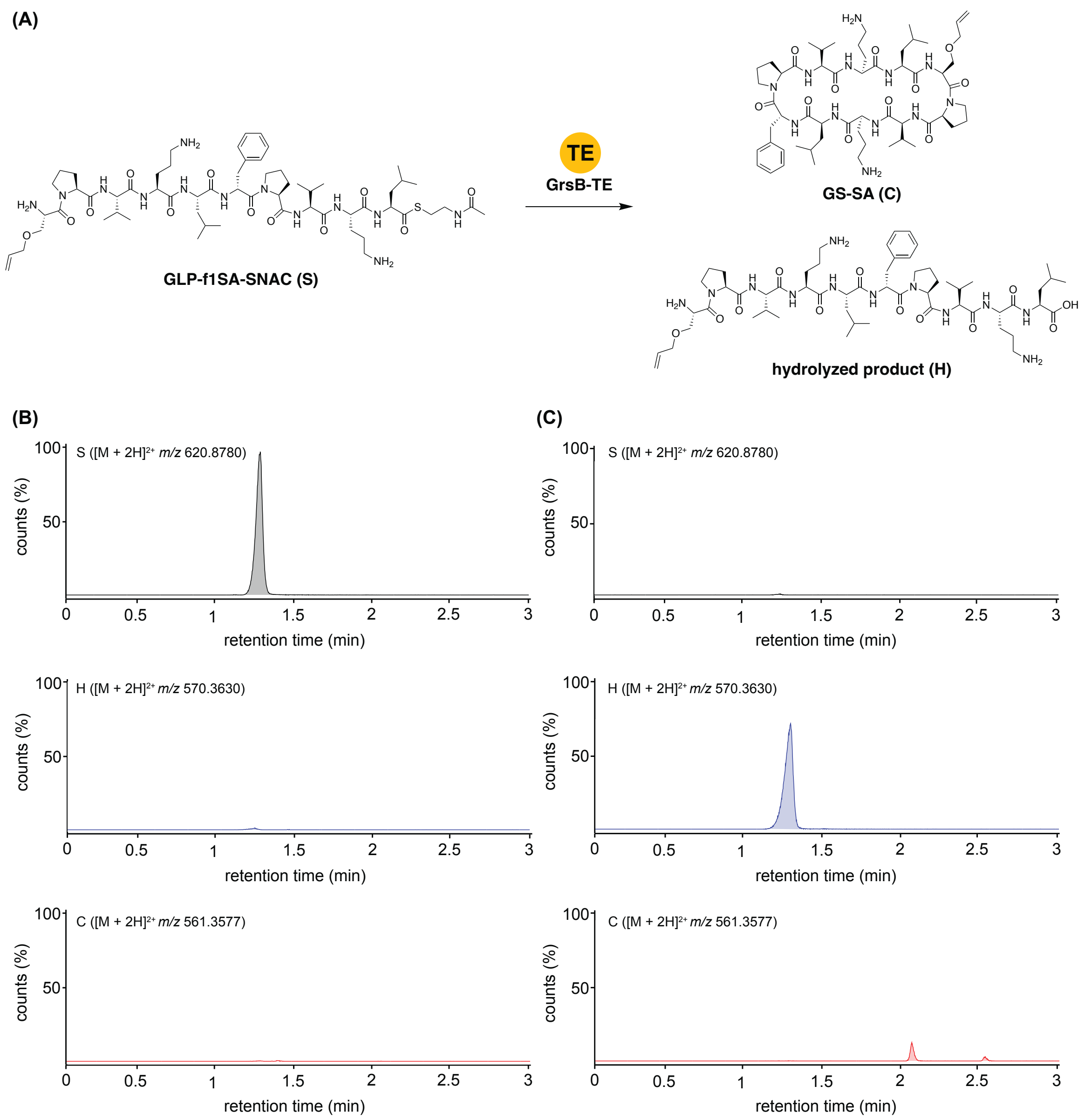

Figure S6. (A) *In vitro* enzymatic assay with GLP-f1SA-SNAC. Separated LC/MS traces of the reactions containing GLP-f1SA-SNAC, without **(B)** or with **(C)** GrsB-TE, corresponding to Figure 2E. The top, middle, and bottom panels show EICs of GLP-f1SA-SNAC (S, $[M + 2H]^{2+}$ *m/z* 620.8780), the hydrolyzed product (H, $[M + 2H]^{2+}$ *m/z* 570.3630), and GS-SA (C, $[M + 2H]^{2+}$ *m/z* 561.3577), respectively.

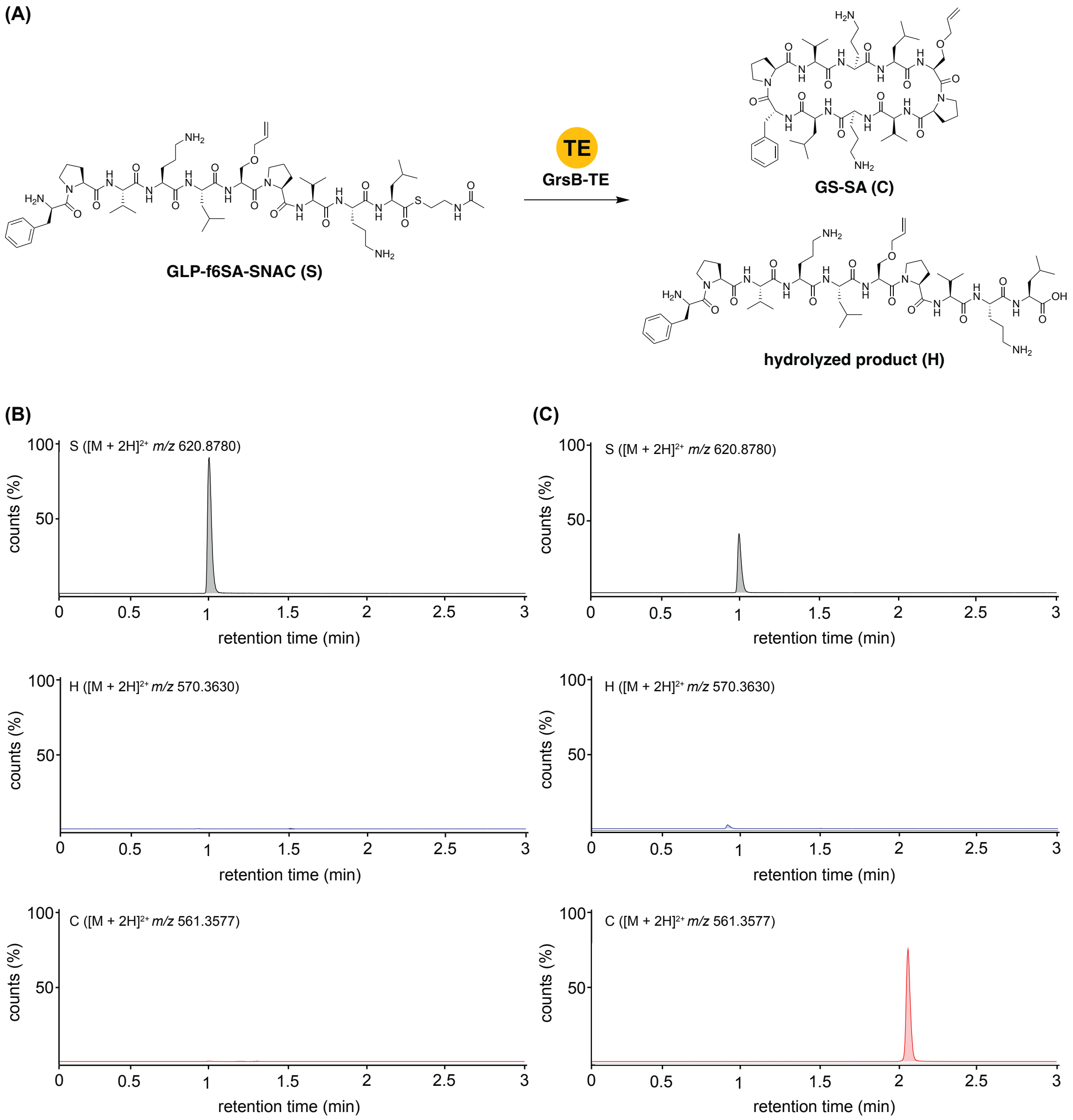

Figure S7. (A) *In vitro* enzymatic assay with GLP-f6SA-SNAC. Separated LC/MS traces of the reactions containing GLP-f6SA-SNAC, without **(B)** or with **(C)** GrsB-TE, corresponding to Figure 2F. The top, middle, and bottom panels show EICs of GLP-f6SA-SNAC (S, $[M + 2H]^{2+}$ *m/z* 620.8780), the hydrolyzed product (H, $[M + 2H]^{2+}$ *m/z* 570.3630), and GS-SA (C, $[M + 2H]^{2+}$ *m/z* 561.3577), respectively.

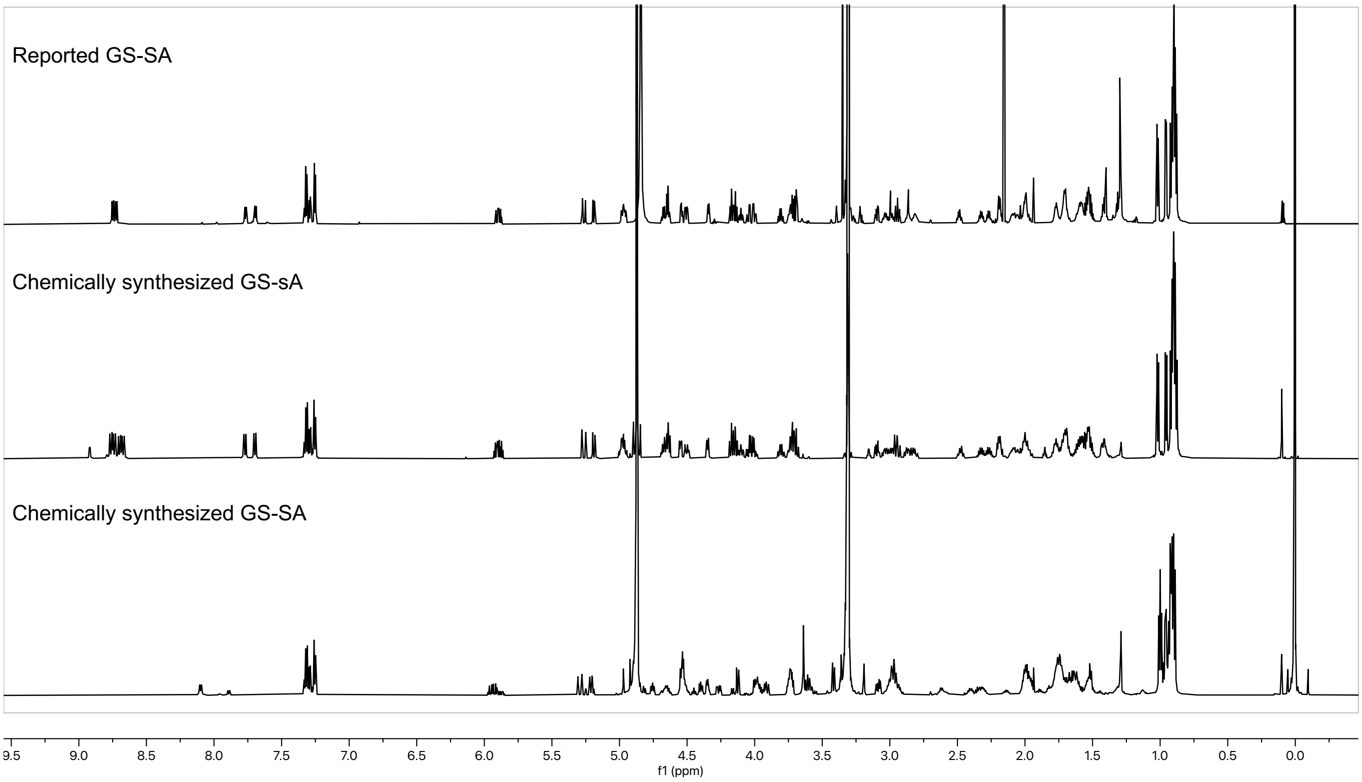

Figure S8. Comparison of the ^{1}H NMR spectra of the reported GS-SA (top), chemically synthesized GS-sA (middle), and chemically synthesized GS-SA (bottom). The top spectrum was generated from the original ^{1}H NMR raw data used in Ref 24, provided by the original authors.

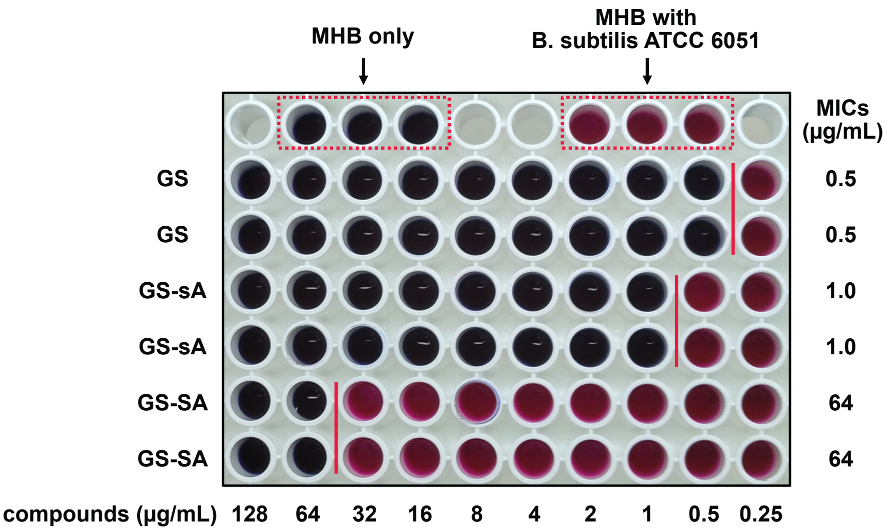

Figure S9. Determination of the minimum inhibitory concentration (MIC) of GS, GS-sA, and GS-SA against *Bacillus subtilis* ATCC 6051. Bacterial growth was assessed using resazurin, a purple dye that is reduced to the pink compound resorufin by live cells. Red lines indicate MIC values in µg/mL.

Biochemical Procedures

Materials

The codon optimized gene containing GrsB-PCP-TE was purchased from Twist Bioscience and inserted into pET28a. The GrsB-TE gene was amplified using Q5 High-Fidelity DNA Polymerase (NEB) with the forward primer 5'-GTAAATGTAGAAGCAGATAGAGAGGCGCTATCTC-3' and reverse primer 5'-CATATGGCTGCCGCGCGG-3'. The PCR products were ligated using KLD mix (NEB) and transformed into *Escherichia coli* NEB-5α (NEB), and the single colony was picked and grown in LB medium (5 mL) with kanamycin (50 µg/mL). The plasmid was extracted by QIAprep spin miniprep kit (Qiagen). The sequence was confirmed by DNA sequencing (Azenta). An authentic sample of GS-SA, as described in Ref 24, was kindly provided by the original authors. This sample was used for the HPLC analysis shown in Figure 3.

Expression and purification of recombinant GrsB-TE

E. coli BL21 (DE3) transformed with the GrsB-TE_pET28a was cultured in LB medium (5 mL x 4) with kanamycin (50 µg/mL) at 37 °C. The overnight culture was inoculated into LB medium (750 mL x 4) with kanamycin (50 µg/mL). The cells were grown at 37 °C until the OD_{600} reached 0.6–0.8, induced by the addition of 0.5 mM isopropyl-*β*-D-thiogalactoside (IPTG), and shaken for 16 h at 16 °C. The cells were harvested by centrifugation at 5000 x g, 4 °C for 5min, and the pellets were resuspended in 60 mL of the lysis buffer (50 mM Tris·HCl, 150 mM NaCl, 10% glycerol, pH 7.4), and then lysed by sonication on ice. The lysates were clarified by centrifugation at 10000 x g at 4 °C for 50 min. The supernatant was incubated with a His-Pur™ Ni-NTA resin (Thermo Scientific), equilibrated with the lysis buffer, at 4°C for 60 min. The resin was washed with the lysis buffer, followed by elution with an imidazole at concentrations ranging from 10 to 400 mM. The fractions containing the target protein were combined and dialyzed with SnakeSkin™ dialysis tubing (10K, Thermo Scientific) into the dialysis buffer (50 mM Tris·HCl, 150 mM NaCl, 10% glycerol, pH 7.4) at 4 °C overnight. The proteins were concentrated by using Amicon Ultra 15 centrifugal filters (10 kDa, Merck Millipore) at 5000 x g at 4°C. The concentrated proteins were stored at –80 °C.

Preparation of peptide stock solutions

Peptides were lyophilized and stored as di-TFA salts. For stock solution preparation, the lyophilized compound was accurately weighed using an analytical balance. The weighed sample was then dissolved in DMSO. The molecular weight used for concentration calculations included two equivalents of TFA per peptide.

Enzymatic assay

GLP-SNAC or its derivatives (200 μM) were incubated with GrsB-TE (10 μM) in 20 mM Tris·HCl buffer (pH 7.0) at 24 °C for 2 h. In all experiments, the final DMSO concentration was maintained at 2%. The reaction (50 μL) was quenched with 2 μL of 4% TFA, followed by the addition of 4 volumes of cold MeOH (208 μL). The sample was incubated on ice for 20 min and then centrifuged at 10,000 × g for 5 min at 4 °C. The resulting supernatant was diluted 10-fold with 80% MeOH and analyzed using an Agilent1290 Infinity II system coupled to a 6530 QTOF MS, equipped with a ZORBAX Eclipse Plus C18 column (2.0 × 50 mm, 1.8 μm particle size). Solvent A was water containing 0.1% formic acid, and solvent B was MeCN containing 0.1% formic acid. The analysis was performed with a linear gradient from 20% to 90% solvent B over 7 min at a flow rate of 0.5 mL/min. The column oven was maintained at 40 °C. Substrates and products were monitored by extracted ion chromatograms (EICs).

QTOF MS setting

Mass spectra were acquired in the positive ion mode over an *m/z* range of 100–1700. The ion source was the Dual Agilent Jet Stream (AJS) ESI. The following instrument parameters were used: gas temperature, 300 °C; drying gas flow, 10 L/min; nebulizer gas (nitrogen) pressure, 60 psig; sheath gas temperature, 350 °C; and sheath gas flow, 12 L/min. The following scan source parameters were used: capillary voltage, 4000 V (positive); nozzle voltage, 500 V; fragmentor voltage, 170 V; skimmer voltage, 65 V; and octopole RF peak voltage, 750 V. MS data were acquired from 0.00 to 5.00 min at a scan rate of 10 spectra/sec.

Determination of MICs of GS analogs against *Bacillus subtilis* ATCC 6051

B. subtilis ATCC 6051 was cultured overnight at 37 °C using a cation-adjusted Mueller Hinton Broth-II (CA-MHB-II) and adjusted to obtain turbidity comparable to 0.5 McFarland standards. The cells were further diluted 1:100 in CA-MHB-II. Two-fold test compound (100 μL, 0.5 to 256 μg/mL) was added to sterile CA-MHB-II in a microtiter plate prior to adding the bacterial suspension. The final DMSO concentration was maintained at 2%. Inoculated and uninoculated wells of compound-free medium were also prepared as the controls. The assay was performed in duplicates. To enhance MIC visualization, resazurin was used as an indicator following a method similar to that of Sarker *et al.*[30] A sterile aqueous resazurin solution (10 μL, 0.7% w/v) was added to each well. After further incubation at 37 °C for 6 h, the plates were photographed.

Measurement of CD spectra for GS analogs

The circular dichroism spectra of the peptide derivatives were measured using a Jasco J-1500CD spectrometer (JASCO, Japan) and a quartz cell with a 0.1 cm path length. Spectra of the peptides dissolved in MeOH were collected between 190 and 260 nm with a scan speed of 100 nm/min, a response time of 1 s, and a bandwidth of 0.1 nm.

Chemical Synthetic Procedures

General Synthetic Methods: Boc-L-Ser(Allyl)-OH[24] was known compound. The compound was prepared according to published literature procedure. Reagents and solvents were purchased from FujiFilm Wako Pure Chemical Industries (Osaka, Japan), Kanto Chemical Co., Inc. (Tokyo, Japan), Sigma-Aldrich (St. Louis, MO), Watanabe Chemical Industries (Hiroshima, Japan), Tokyo Chemical Industries (Tokyo, Japan), and Nacalai tesque (Kyoto, Japan). All chemical reagents were used as received. Column chromatography was performed on silica gel 60 N (spherical, neutral; 40–50 µm) and thin-layer chromatography (TLC) was performed on precoated plates (0.25 mm, silica gel Merck Kieselgel 60F245). ^{1}H NMR spectra were measured in $CDCl_3$, methanol-*d*$_4$, or DMSO-*d*$_6$ solution and referenced to TMS (0.00 ppm) using Bruker DPX-400 (400 MHz) and JEOL JNM-ECZ600R (600 MHz) NMR spectrometers. ^{13}C NMR spectra were measured in $CDCl_3$, methanol-*d*$_4$, or DMSO-*d*$_6$ solution and referenced to a residual solvent peak of $CDCl_3$ (77.05 ppm), methanol-*d*$_4$ (49.00 ppm), or DMSO-*d*$_6$ (39.52 ppm) using a Bruker DPX-400 (100 MHz) and JEOL JNM-ECZ600R (150 MHz) NMR spectrometers. Chemical shift δ values for ^{1}H and ^{13}C spectra are reported in parts per million (ppm) relative to these referenced values, and multiplicities are abbreviated as s = singlet, d = doublet, t = triplet, q = quartet, m = multiplet, br = broad. All ^{13}C NMR spectra were recorded with complete proton decoupling. FID files were processed using Mnova NMR (Mestrelab research). High resolution mass spectra were obtained on a Waters MICRO MASS LCT-premier (ESI).

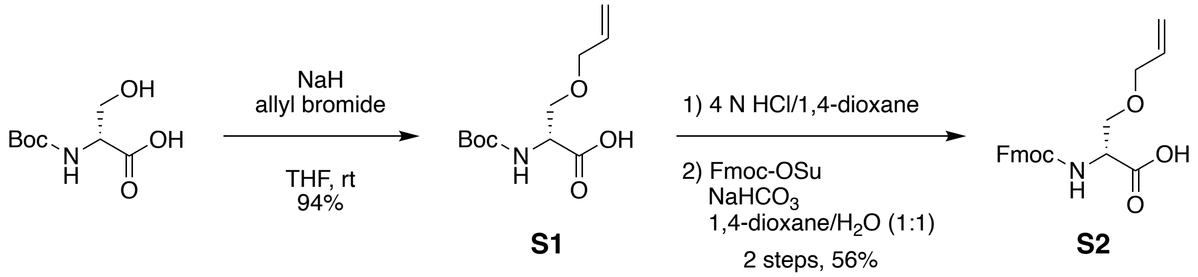

Scheme S1. Synthesis of Fmoc-D-Ser(Allyl)-OH.

O-Allyl-*N*-(*tert*-butoxycarbonyl)-*D*-serine (S1)

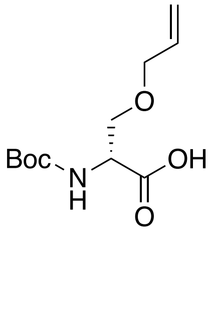

NaH (487 mg, 12.2 mmol) was slowly added to a solution of Boc-D-Ser-OH (1.00 g, 4.87 mmol) in DMF (25 mL) at 0 °C. The mixture was stirred at 0 °C for 30 min, then allyl bromide (464 µL, 5.36 mmol) was added. After stirring at rt for 1 h, the reaction was quenched with water (5 mL), and DMF was evaporated. The residue was washed with EtOAc and acidified using solid citric acid monohydrate until the pH reached at 3–4. The aqueous phase was extracted with EtOAc, and the combined organic layer was washed with sat. NaCl, dried over Na_2SO_4, and concentrated to afford compound **S1** (1.13 g, 94%) as a slightly yellow oil.

^{1}H NMR (600 MHz, methanol-*d*$_4$) δ 5.95 – 5.82 (m, 1H), 5.27 (dd, *J* = 17.3, 1.7 Hz, 1H), 5.16 (dd, *J* = 10.5, 1.6 Hz, 1H), 4.31 (t, *J* = 4.2 Hz, 1H), 4.07 – 3.94 (m, 2H), 3.80 (dd, *J* = 9.7, 4.8 Hz, 1H), 3.68 (dd, *J* = 9.7, 3.8 Hz, 1H), 1.45 (s, 9H). ^{13}C NMR (150 MHz, methanol-*d*$_4$) δ 173.7, 157.8, 135.6, 117.5, 80.7, 73.1, 70.8, 55.2, 28.7. HR-ESI-MS calcd. $C_{11}H_{19}NO_5Na$, $[M+Na]^+$: 268.1161, found 268.1159.

***N*-(((9*H*-fluoren-9-yl)methoxy)carbonyl)-*O*-allyl-*D*-serine (S2)**

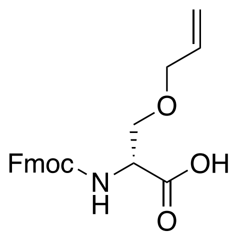

Compound **S1** (619 mg, 2.52 mmol) was dissolved in 4 N HCl/1,4-dioxane (5.0 mL) and stirred at rt for 30 min. The solvent was then evaporated to dryness. The residue was dissolved in 1,4-dioxane/water (9 mL, 1:1), and Fmoc-OSu (809 mg, 2.40 mmol) and $NaHCO_3$ (636 mg, 7.57 mmol) were added at 0 °C. After stirring at rt for 3 h, the reaction was diluted with EtOAc, washed with 1 N HCl and sat. NaCl, and dried over Na_2SO_4, and concentrated. The residue was purified by flash silica-gel chromatography ($CHCl_3$/MeOH = 10:1) to afford compound **S2** (515 mg, 56%) as a white solid.

^{1}H NMR (600 MHz, DMSO-*d*$_6$) δ 7.85 (d, *J* = 7.6 Hz, 2H), 7.70 (d, *J* = 7.5 Hz, 2H), 7.38 (t, *J* = 7.5 Hz, 2H), 7.30 (t, *J* = 7.5 Hz, 2H), 7.05 (d, *J* = 8.1 Hz, 1H), 5.89 – 5.76 (m, 1H), 5.21 (d, *J* = 17.3 Hz, 1H), 5.07 (dd, *J* = 10.2, 2.1 Hz, 1H), 4.38 – 4.29 (m, 1H), 4.27 – 4.17 (m, 2H), 4.20 – 4.11 (m, 1H), 3.97 – 3.88 (m, 2H), 3.76 – 3.63 (m, 2H). ^{13}C NMR (150 MHz, DMSO-*d*$_6$) δ 174.3, 155.8, 144.1, 144.0, 140.8, 140.8, 135.4, 127.7, 127.2, 125.4, 125.3, 120.2, 116.3, 71.1, 70.4, 65.7, 55.8, 46.8. HR-ESI-MS calcd. $C_{21}H_{21}NO_5Na$, $[M+Na]^+$: 390.1317, found 390.1312.

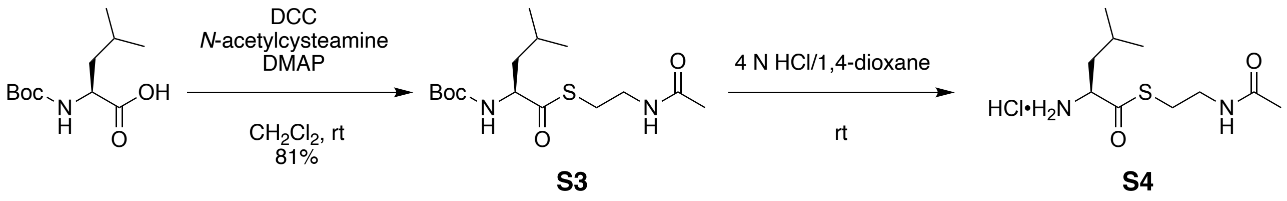

Scheme S2. Synthesis of Boc-L-Leu-SNAC.

S-(2-Acetamidoethyl) (S)-2-((tert-butoxycarbonyl)amino)-4-methylpentanethioate (S3)

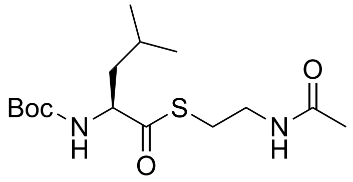

DCC (196 mg, 0.95 mmol) was added to a solution of Boc-L-Leu-OH (200 mg, 0.87 mmol), *N*-acetylcysteamine (155 mg, 1.30 mmol), and DMAP (10.6 mg, 0.087 mmol) in CH_2Cl_2 at rt. The mixture was stirred for 3 h at rt, and then filtered with a pipette filter with a small piece of cotton to remove the precipitated byproduct. The filtrate was evaporated to dryness. The residue was purified by MPLC (Biotage) (8–66% EtOAc in hexane) to afford 232 mg (81%) of compound **S3** as a white amorphous.

^{1}H NMR (400 MHz, $CDCl_3$) δ 6.08 (s, 1H), 5.00 (d, J = 8.2 Hz, 1H), 4.37 – 4.27 (m, 1H), 3.53 – 3.30 (m, 2H), 3.11 – 2.96 (m, 2H), 1.96 (s, 3H), 1.80 – 1.56 (m, 2H), 1.50 – 1.43 (m, 10H), 0.96 (d, J = 5.0 Hz, 3H), 0.94 (d, J = 4.9 Hz, 3H). ^{13}C NMR (100 MHz, $CDCl_3$) δ 202.7, 170.5, 155.4, 80.4, 59.5, 41.4, 39.3, 28.3, 28.2, 24.8, 23.1, 23.0, 21.5.

HR-ESI-MS calcd. $C_{15}H_{29}N_2O_4NaS$, $[M+Na]^+$: 355.1667, found 355.1662.

S-(2-Acetamidoethyl) (S)-2-amino-4-methylpentanethioate hydrochloride (S4)

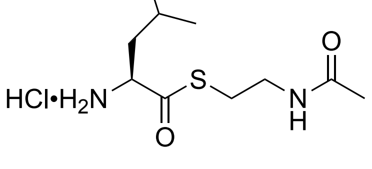

Compound **S3** (200 mg, 0.60 mmol) was dissolved in 4 N HCl/1,4-dioxane (2.0 mL) and stirred at

rt for 30 min. The solvent was evaporated and dried *in vacuo*, and the resulting residue was used in the next step without further purification.

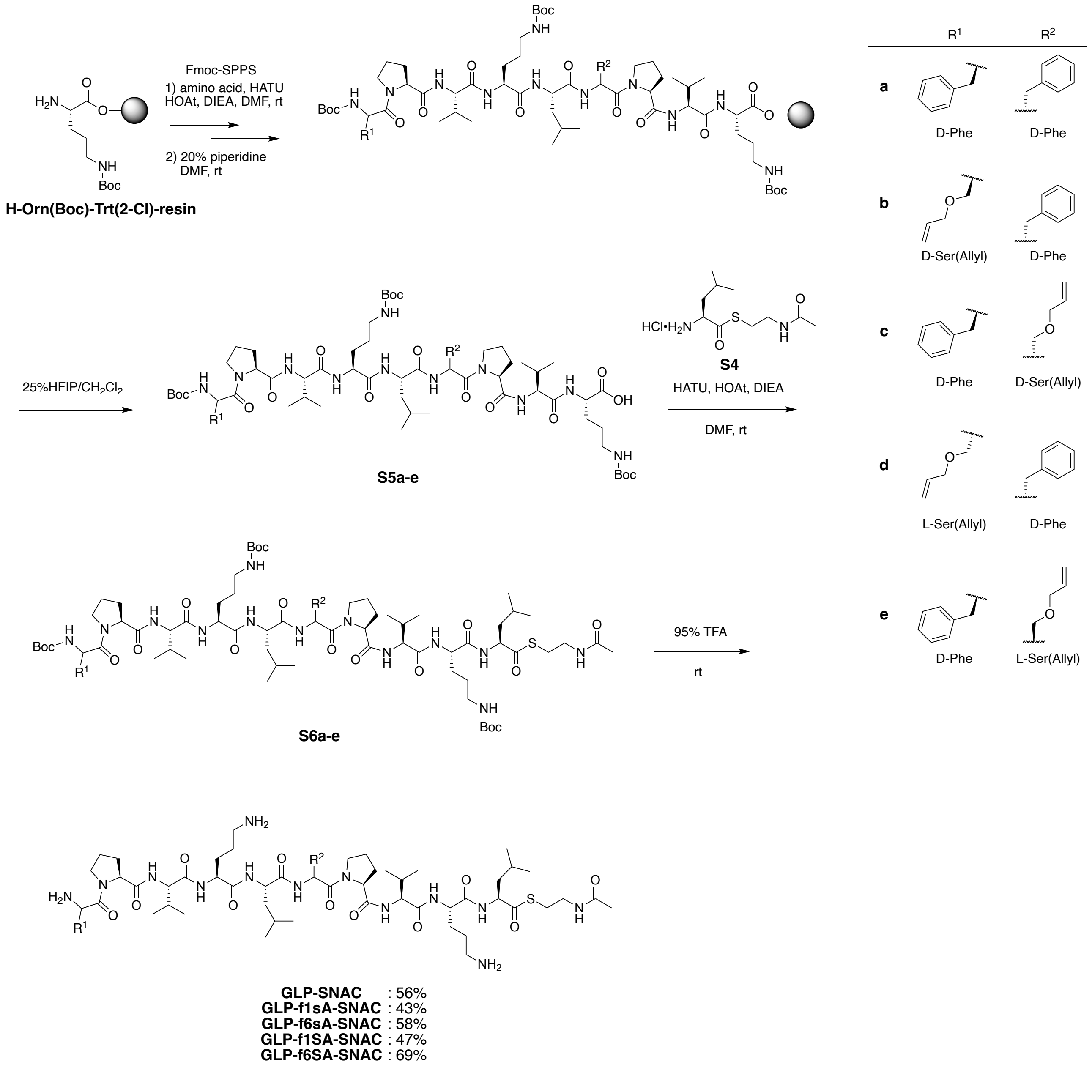

Scheme S3. Synthesis of linear peptide-SNAC substrates.

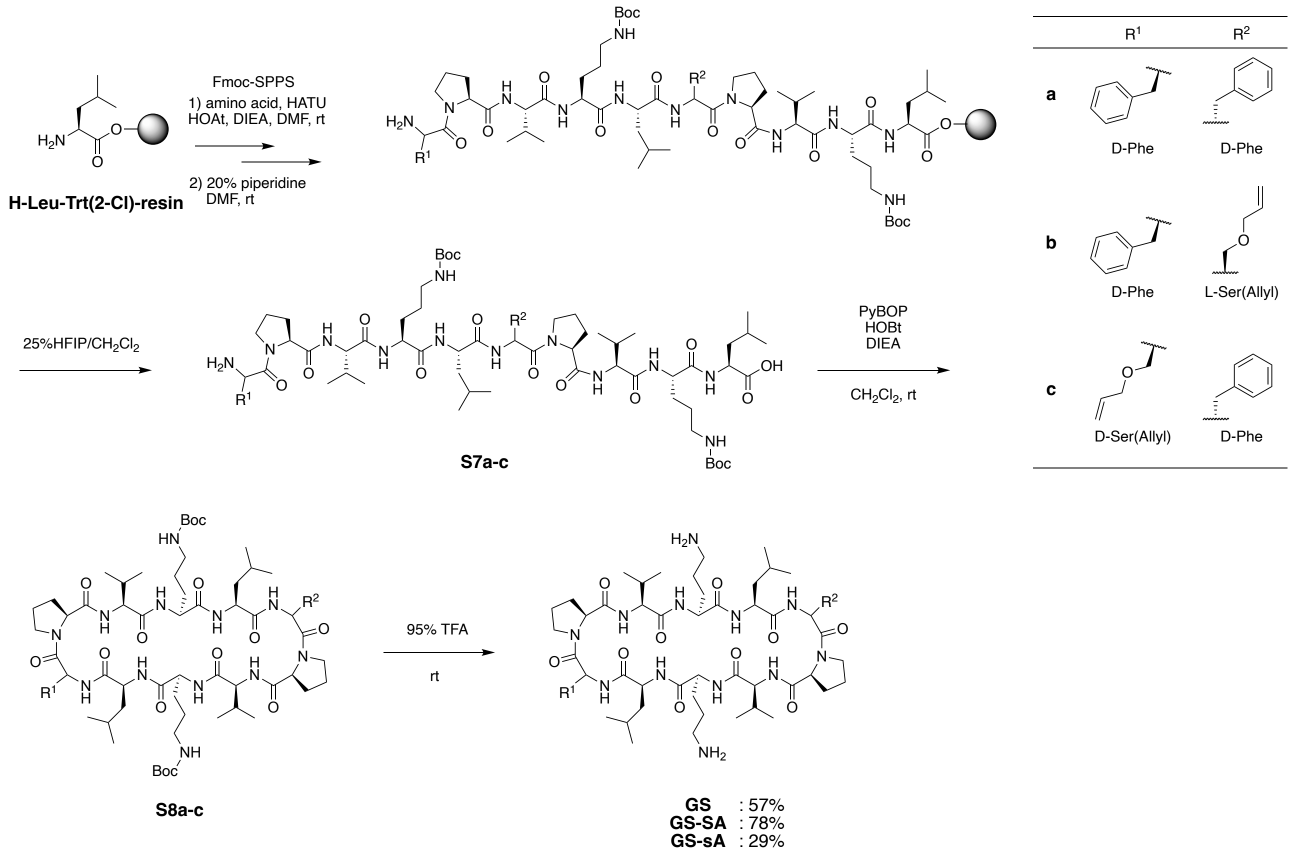

Scheme S4. Synthesis of GS, GS-SA, and GS-sA.

Preparation of H-L-Orn(Boc)-Trt(2-Cl)-resin

H-L-Orn(Boc)-Trt(2-Cl)-resin was prepared as previously described.[31] Briefly, Cl-Trt(2-Cl) resin (962 mg, 1.25 mmol, 1.3 mmol/g, 200-400 mesh, 1% DVB) was swollen with anhydrous CH_2Cl_2 (2 mL) under Ar for 10 min. Fmoc-L-Orn(Boc)-OH (568 mg, 0.75 mmol) and DIEA (522 μL, 3.00 mmol) were mixed with the resin. The mixture was stirred at rt for 1 h and replaced to the polypropylene column tube equipped with polyethylene frits, and the solvent was removed *in vacuo*. The resin was shaken with pre-mixed CH_2Cl_2/MeOH/DIEA (17:2:1) for 10 min. The resin was washed with CH_2Cl_2 x 3, DMF x 3, CH_2Cl_2 x 3, MeOH, and then dried *in vacuo*. For determination of the loading rate, quantification of dibenzofulvene-piperidine adduct (λ = 301 nm, ε = 7,800 M^{-1} cm^{-1}) derived from the deprotection of Fmoc group with 50% piperidine in DMF was performed with a small amount of the resin. Fmoc group of the residual resin was deprotected with 20% piperidine in DMF, and the resin was washed with DMF and CH_2Cl_2. The dried resin was stored at -20 °C.

General procedures of Fmoc-solid phase peptide synthesis

H-L-Orn(Boc)-Trt(2-Cl)-resin (20–55 μmol) or H-L-Leu-Trt(2-Cl)-resin (40 μmol, Watanabe Chemical Industry) was swollen in DMF for 30 min with gentle agitation. For peptide elongation, the protected amino acid (5 eq.) was coupled to the resin in the presence of HATU (5 eq.), HOAt (5 eq.), and DIEA (10 eq.) at rt for 1 h. Of note, double coupling was performed for the coupling of protected amino acids after the incorporation of Pro. The Fmoc group was deprotected using 20% piperidine in DMF at rt for 20 min. Reaction completion was monitored by Kaiser test. The elongated protected peptides were cleaved from the resin using 20% HFIP in CH_2Cl_2 at rt for 15 min, then dried under a stream of N_2 gas to obtain the protected peptides **S5a-e** or **S7a-c**.

GLP-SNAC

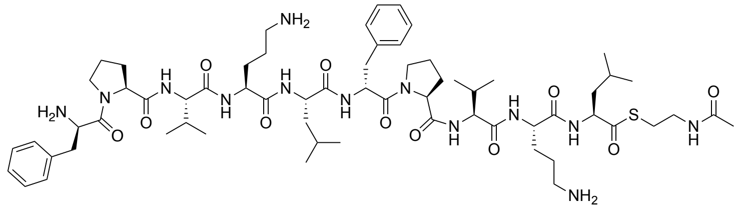

Crude **S5a** was prepared starting from H-L-Orn(Boc)-Trt(2-Cl)-resin (0.055 mmol). HATU (42 mg, 0.11 mmol) and HOAt (15 mg, 0.11 mmol) were added to a solution of compound **S4** (15 mg, 0.066 mmol) and **S5a** (ca. 0.055 mmol) in DMF at rt. DIEA was then added dropwise until the pH reached 7–8. The mixture was stirred at rt for 30 min and then evaporated to dryness. The resulting residue containing compound **S6a** was dissolved in 95% aqueous TFA and stirred at rt for 3 h. The solvent was then removed under a stream of N_2 gas, and the resulting pellet was washed three times with cold Et_2O. The obtained white solid was dissolved in 50% $MeCN/H_2O$ and purified by preparative RP-HPLC using SunFire Prep C18 OBD^{TM} (19 × 150 mm, 5 μm). Solvent A was water containing 0.1% TFA, and solvent B was MeCN containing 0.1% TFA. The purification was performed using a linear gradient from 30% to 50% solvent B over 20 min at a flow rate of 5.0 mL/min. The elution was monitored at 220 nm. **GLP-SNAC** was obtained as a white amorphous solid.

The purity was determined by analytical RP-HPLC using COSMOSIL C_{18} AR-II (4.6 × 150 mm, 5 μm). Solvent A was water containing 0.1% TFA, and solvent B was MeCN containing 0.1% TFA. The purification was performed using a linear gradient from 10% to 50% solvent B over 40 min at a flow rate of 1.0 mL/min. The analysis was monitored at 220 nm.

Yield: 39.8 mg (56%, calculated as the 2TFA salt). Purity: 98.4%. HRMS (ESI+): $[M+H]^+$ calcd. for $C_{64}H_{102}N_{13}O_{11}S$, 1260.7542; found, 1260.7538.

GLP-f1sA-SNAC

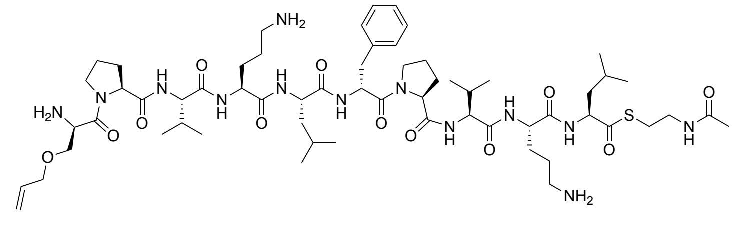

Crude **S5b** was prepared starting from H-L-Orn(Boc)-Trt(2-Cl)-resin (0.026 mmol). HATU (20 mg, 0.053 mmol) and HOAt (7.2 mg, 0.053 mmol) were added to a solution of compound **S4** (7.4 mg, 0.032 mmol) and **S5b** (ca. 0.026 mmol) in DMF at rt. DIEA was then added dropwise until the pH reached 7–8. The mixture was stirred at rt for 30 min and then evaporated to dryness. The resulting residue containing compound **S6b** was dissolved in 95% aqueous TFA and stirred at rt for 3 h. The solvent was then removed under a stream of N_2 gas, and the resulting pellet was washed three times with cold Et_2O. The obtained white solid was dissolved in 50% MeCN/H_2O and purified by preparative RP-HPLC using SunFire Prep C18 OBD^{TM} (19 × 150 mm, 5 μm). Solvent A was water containing 0.1% TFA, and solvent B was MeCN containing 0.1% TFA. The purification was performed using a linear gradient from 30% to 45% solvent B over 15 min at a flow rate of 5.0 mL/min. The elution was monitored at 220 nm. **GLP-f1sA-SNAC** was obtained as a white amorphous solid.

The purity was determined by analytical RP-HPLC using COSMOSIL C_{18} AR-II (4.6 × 150 mm, 5 μm). Solvent A was water containing 0.1% TFA, and solvent B was MeCN containing 0.1% TFA. The analysis was performed using a linear gradient from 10% to 50% solvent B over 40 min at a flow rate of 1.0 mL/min, with detection at 220 nm.

Yield: 15.1 mg (43%, calculated as the 2TFA salt). Purity: 99.8%. HRMS (ESI+): $[M+H]^+$ calcd. for $C_{61}H_{102}N_{13}O_{12}S$, 1240.7492; found, 1240.7490.

GLP-f6sA-SNAC

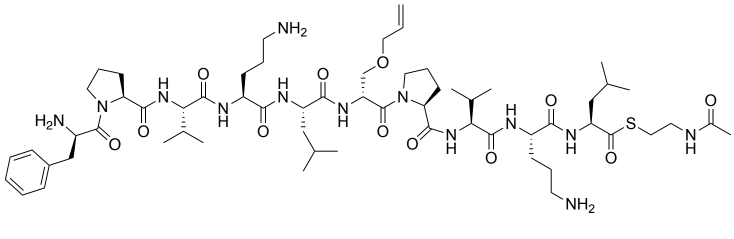

Crude **S5c** was prepared starting from H-L-Orn(Boc)-Trt(2-Cl)-resin (0.020 mmol). HATU (15 mg, 0.040 mmol) and HOAt (5.4 mg, 0.040 mmol) were added to a solution of compound **S4** (5.6 mg,

0.024 mmol) and **S5c** (ca. 0.020 mmol) in DMF at rt. DIEA was then added dropwise until the pH reached 7–8. The mixture was stirred at rt for 30 min and then evaporated to dryness. The resulting residue containing compound **S6c** was dissolved in 95% aqueous TFA and stirred at rt for 3 h. The solvent was then removed under a stream of N_2 gas, and the resulting pellet was washed three times with cold Et_2O. The obtained white solid was dissolved in 50% MeCN/H_2O and purified by preparative RP-HPLC using SunFire Prep C18 OBD^{TM} (19 × 150 mm, 5 μm). Solvent A was water containing 0.1% TFA, and solvent B was MeCN containing 0.1% TFA. The purification was performed using a linear gradient from 30% to 50% solvent B over 20 min at a flow rate of 5.0 mL/min. The elution was monitored at 220 nm. **GLP-f6sA-SNAC** was obtained as a white amorphous solid.

The purity was determined by analytical RP-HPLC using COSMOSIL C_{18} AR-II (4.6 × 150 mm, 5 μm). Solvent A was water containing 0.1% TFA, and solvent B was MeCN containing 0.1% TFA. The analysis was performed using a linear gradient from 10% to 50% solvent B over 40 min at a flow rate of 1.0 mL/min, with detection at 220 nm.

Yield: 14.7 mg (58%, calculated as the 2TFA salt). Purity: 99.1%. HRMS (ESI+): $[M+H]^+$ calcd. for $C_{61}H_{102}N_{13}O_{12}S$, 1240.7492; found, 1240.7489.

GLP-f1SA-SNAC

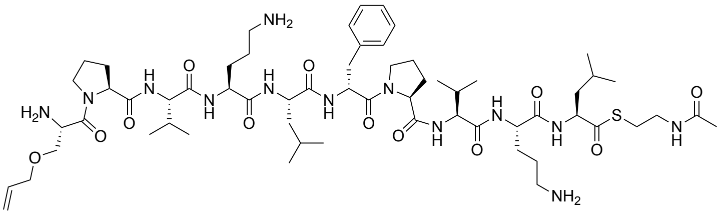

Crude **S5d** was prepared starting from H-L-Orn(Boc)-Trt(2-Cl)-resin (0.029 mmol). HATU (22 mg, 0.057 mmol) and HOAt (7.8 mg, 0.057 mmol) were added to a solution of compound **S4** (8.0 mg, 0.034 mmol) and **S5d** (ca. 0.029 mmol) in DMF at rt. DIEA was then added dropwise until the pH reached 7–8. The mixture was stirred at rt for 30 min and then evaporated to dryness. The resulting residue containing compound **S6d** was dissolved in 95% aqueous TFA and stirred at rt for 3 h. The solvent was then removed under a stream of N_2 gas, and the resulting pellet was washed three times with cold Et_2O. The obtained white solid was dissolved in 50% MeCN/H_2O and purified by preparative RP-HPLC using SunFire Prep C18 OBD^{TM} (19 × 150 mm, 5 μm). Solvent A was water containing 0.1% TFA, and solvent B was MeCN containing 0.1% TFA. The purification was performed using a linear gradient from 30% to 45% solvent B over 15 min at a flow rate of 5.0 mL/min. The elution was monitored at 220 nm. **GLP-f1SA-SNAC** was obtained as a white amorphous solid.

The purity was determined by analytical RP-HPLC using COSMOSIL C_{18} AR-II (4.6 × 150 mm, 5

μm). Solvent A was water containing 0.1% TFA, and solvent B was MeCN containing 0.1% TFA. The analysis was performed using a linear gradient from 10% to 50% solvent B over 40 min at a flow rate of 1.0 mL/min, with detection at 220 nm.
Yield: 16.8 mg (47%, calculated as the 2TFA salt). Purity: 98.9%. HRMS (ESI+): $[M+H]^+$ calcd. for $C_{61}H_{102}N_{13}O_{12}S$, 1240.7492; found, 1240.7496.

GLP-f6SA-SNAC

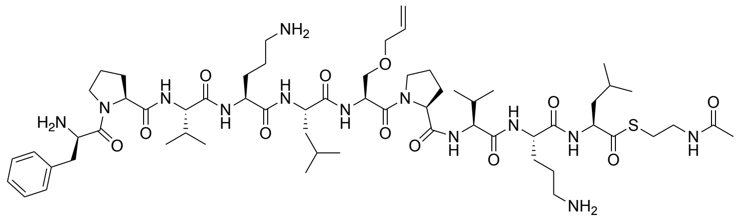

Crude **S5e** was prepared starting from H-L-Orn(Boc)-Trt(2-Cl)-resin (0.040 mmol). HATU (30 mg, 0.080 mmol) and HOAt (11 mg, 0.080 mmol) were added to a solution of compound **S4** (11 mg, 0.048 mmol) and **S5e** (ca. 0.040 mmol) in DMF at rt. DIEA was then added dropwise until the pH reached 7–8. The mixture was stirred at rt for 30 min and then evaporated to dryness. The resulting residue containing compound **S6e** was dissolved in 95% aqueous TFA and stirred at rt for 3 h. The solvent was then removed under a stream of N_2 gas, and the resulting pellet was washed three times with cold Et_2O. The obtained white solid was dissolved in 50% MeCN/H_2O and purified by preparative RP-HPLC using SunFire Prep C18 OBD™ (19 × 150 mm, 5 μm). Solvent A was water containing 0.1% TFA, and solvent B was MeCN containing 0.1% TFA. The purification was performed using a linear gradient from 28% to 43% solvent B over 15 min at a flow rate of 5.0 mL/min. The elution was monitored at 220 nm. **GLP-f6SA-SNAC** was obtained as a white amorphous solid.
The purity was determined by analytical RP-HPLC using COSMOSIL C_{18} AR-II (4.6 × 150 mm, 5 μm). Solvent A was water containing 0.1% TFA, and solvent B was MeCN containing 0.1% TFA. The analysis was performed using a linear gradient from 10% to 50% solvent B over 40 min at a flow rate of 1.0 mL/min, with detection at 220 nm.
Yield: 34.5 mg (69%, calculated as the 2TFA salt). Purity: 99.9%. HRMS (ESI+): $[M+H]^+$ calcd. for $C_{61}H_{102}N_{13}O_{12}S$, 1240.7492; found, 1240.7496.

Gramicidin S

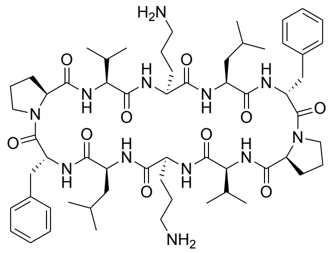

Crude **S7a** was prepared starting from H-L-Leu-Trt(2-Cl)-resin (0.040 mmol). PyBOP (62 mg, 0.12 mmol) and HOBt (18 mg, 0.12 mmol) were added to a solution of compound **S7a** (ca. 0.040 mmol) in CH_2Cl_2 (150 mL) at rt. DIEA (41 µL, 0.24 mmol) was then added dropwise to the mixture, which was stirred at rt for 16 h and then evaporated to dryness. The resulting residue containing compound **S8a** was dissolved in 95% aqueous TFA and stirred at rt for 3 h. The solvent was then removed under a stream of N_2 gas. The residue was dissolved in 50% MeCN/H_2O and purified by preparative RP-HPLC using SunFire Prep C18 OBD™ (19 × 150 mm, 5 µm). Solvent A was water containing 0.1% TFA, and solvent B was MeCN containing 0.1% TFA. The purification was performed using a linear gradient from 45% to 65% solvent B over 20 min at a flow rate of 5.0 mL/min. The elution was monitored at 220 nm. **Gramicidin S** was obtained as a white amorphous solid.

The purity was determined by analytical RP-HPLC using COSMOSIL C_{18} AR-II (4.6 × 150 mm, 5 µm). Solvent A was water containing 0.1% TFA, and solvent B was MeCN containing 0.1% TFA. The analysis was performed using a linear gradient from 30% to 70% solvent B over 40 min at a flow rate of 1.0 mL/min, with detection at 220 nm.

^{1}H NMR (600 MHz, methanol-*d*$_4$) δ 8.92 (d, *J* = 3.8 Hz, 1H), 8.74 (d, *J* = 9.3 Hz, 2H), 8.69 (d, *J* = 9.4 Hz, 2H), 7.70 (d, *J* = 9.2 Hz, 2H), 7.36 – 7.24 (m, 10H), 5.02 – 4.95 (m, 2H), 4.70 – 4.63 (m, 2H), 4.53 – 4.47 (m, 2H), 4.38 – 4.32 (m, 2H), 4.15 (t, *J* = 8.7 Hz, 2H), 3.77 – 3.70 (m, 2H), 3.09 (dd, *J* = 12.7, 4.9 Hz, 2H), 3.07 – 3.00 (m, 2H), 2.94 (t, *J* = 12.0 Hz, 2H), 2.91 – 2.84 (m, 2H), 2.51 – 2.44 (m, 2H), 2.31 – 2.23 (m, 2H), 2.09 – 1.96 (m, 4H), 1.81 – 1.74 (m, 4H), 1.74 – 1.62 (m, 4H), 1.65 – 1.49 (m, 6H), 1.44 – 1.37 (m, 2H), 0.96 (d, *J* = 6.5 Hz, 6H), 0.92 – 0.86 (m, 18H).

Yield: 26.1 mg, (57%, calculated as the 2TFA salt). Purity: 99.9%. HRMS (ESI+): $[M+H]^+$ calcd. for $C_{60}H_{93}N_{12}O_{10}$, 1141.7138; found, 1141.7162.

Gramicidin S-L-Ser(Allyl) (GS-SA)

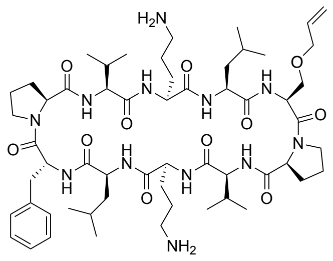

Crude **S7b** was prepared starting from H-L-Leu-Trt(2-Cl)-resin (0.040 mmol). PyBOP (62 mg, 0.12 mmol) and HOBt (18 mg, 0.12 mmol) were added to a solution of compound **S7c** (ca. 0.040 mmol) in CH_2Cl_2 (120 mL) at rt. DIEA (41 µL, 0.24 mmol) was then added dropwise to the mixture, which was stirred at rt for 16 h and then evaporated to dryness. The resulting residue containing compound **S8b** was dissolved in 95% aqueous TFA and stirred at rt for 3 h. The solvent was then removed under a stream of N_2 gas. The residue was dissolved in 50% MeCN/H_2O and purified by preparative RP-HPLC using SunFire Prep C18 OBD^{TM} (19 × 150 mm, 5 µm). Solvent A was water containing 0.1% TFA, and solvent B was MeCN containing 0.1% TFA. The purification was performed using a linear gradient from 35% to 55% solvent B over 20 min at a flow rate of 5.0 mL/min. The elution was monitored at 220 nm. **GS-SA** was obtained as a white amorphous solid.

The purity was determined by analytical RP-HPLC using COSMOSIL C_{18} AR-II (4.6 × 150 mm, 5 µm). Solvent A was water containing 0.1% TFA, and solvent B was MeCN containing 0.1% TFA. The analysis was performed using a linear gradient from 30% to 70% solvent B over 40 min at a flow rate of 1.0 mL/min, with detection at 220 nm.

^{1}H NMR (600 MHz, methanol-*d*$_4$) δ 7.37 – 7.22 (m, 5H), 6.01 – 5.81 (m, 1H), 5.34 – 5.22 (m, 1H), 5.25 – 5.16 (m, 1H), 4.76 (dd, *J* = 8.1, 5.9 Hz, 1H), 4.72 – 4.59 (m, 1H), 4.53 (tt, *J* = 9.7, 4.6 Hz, 4H), 4.40 (dd, *J* = 8.6, 5.7 Hz, 1H), 4.35 (d, *J* = 6.4 Hz, 1H), 4.27 (dd, *J* = 12.8, 5.4 Hz, 1H), 4.14 (dd, *J* = 23.1, 8.8 Hz, 1H), 4.04 – 3.87 (m, 2H), 3.79 – 3.68 (m, 2H), 3.68 – 3.54 (m, 2H), 3.09 (dd, *J* = 12.6, 5.5 Hz, 1H), 3.07 – 2.87 (m, 5H), 2.67 – 2.54 (m, 1H), 2.48 – 2.26 (m, 2H), 2.15 (ddd, *J* = 21.6, 16.7, 8.3 Hz, 1H), 2.05 – 1.90 (m, 6H), 1.86 – 1.59 (m, 13H), 1.60 – 1.46 (m, *J* = 6.8 Hz, 2H), 1.04 – 0.86 (m, 24H).

Yield: 42.1 mg, (78%, calculated as the 2TFA salt). Purity: 96.5%. $[M+H]^+$ calcd. for $C_{57}H_{93}N_{12}O_{11}$, 1121.7087; found, 1121.7076.

Gramicidin S-D-Ser(Allyl) (GS-sA)

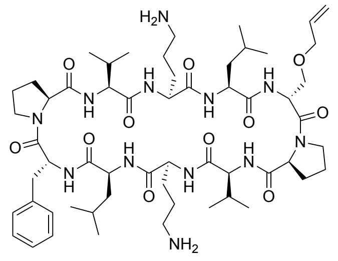

Crude **S7c** was prepared starting from H-L-Leu-Trt(2-Cl)-resin (0.040 mmol). PyBOP (62 mg, 0.12 mmol) and HOBt (18 mg, 0.12 mmol) were added to a solution of compound **S7c** (ca. 0.040 mmol) in CH_2Cl_2 (120 mL) at rt. DIEA (41 µL, 0.24 mmol) was then added dropwise to the mixture, which was stirred at rt for 16 h and then evaporated to dryness. The resulting residue containing compound **S8c** was dissolved in 95% aqueous TFA and stirred at rt for 3 h. The solvent was then removed under a stream of N_2 gas. The residue was dissolved in 50% MeCN/H_2O and purified by preparative RP-HPLC using SunFire Prep C18 OBD^{TM} (19 × 150 mm, 5 µm). Solvent A was water containing 0.1% TFA, and solvent B was MeCN containing 0.1% TFA. The purification was performed using a linear gradient from 50% to 65% solvent B over 15 min at a flow rate of 5.0 mL/min. The elution was monitored at 220 nm. **GS-sA** was obtained as a white amorphous solid.

The purity was determined by analytical RP-HPLC using COSMOSIL C_{18} AR-II (4.6 × 150 mm, 5 µm). Solvent A was water containing 0.1% TFA, and solvent B was MeCN containing 0.1% TFA. The analysis was performed using a linear gradient from 30% to 70% solvent B over 40 min at a flow rate of 1.0 mL/min, with detection at 220 nm.

^{1}H NMR (600 MHz, methanol-*d*$_4$) δ 8.75 (dd, *J* = 15.2, 9.4 Hz, 2H), 8.68 (dd, *J* = 16.2, 9.3 Hz, 2H), 7.77 (d, *J* = 8.9 Hz, 1H), 7.70 (d, *J* = 9.0 Hz, 1H), 7.37 – 7.22 (m, 5H), 5.96 – 5.83 (m, 1H), 5.26 (dd, *J* = 17.4, 1.7 Hz, 1H), 5.19 (dd, *J* = 10.0, 1.5 Hz, 1H), 5.03 – 4.92 (m, 2H), 4.72 – 4.60 (m, 3H), 4.55 (dd, *J* = 7.0, 3.5 Hz, 1H), 4.50 (dd, *J* = 11.4, 4.9 Hz, 1H), 4.39 – 4.31 (m, 1H), 4.17 (t, *J* = 8.6 Hz, 1H), 4.14 (d, *J* = 8.9 Hz, 1H), 4.15 – 4.05 (m, 1H), 4.09 – 3.95 (m, 2H), 3.86 – 3.75 (m, 1H), 3.79 – 3.65 (m, 3H), 3.09 (dd, *J* = 12.6, 4.9 Hz, 1H), 3.09 – 2.95 (m, 2H), 2.99 – 2.89 (m, 1H), 2.92 – 2.76 (m, 2H), 2.53 – 2.42 (m, 1H), 2.38 – 2.21 (m, 2H), 2.24 – 2.14 (m, 2H), 2.15 – 1.91 (m, 5H), 1.83 – 1.64 (m, 6H), 1.68 – 1.46 (m, 7H), 1.47 – 1.35 (m, 2H), 1.02 (d, *J* = 6.7 Hz, 3H), 0.96 (d, *J* = 6.8 Hz, 3H), 0.95 – 0.84 (m, 18H).

Yield: 13.1 mg, (29%, calculated as the 2TFA salt). Purity: 99.3%. HRMS (ESI+): $[M+H]^+$ calcd. for $C_{57}H_{93}N_{12}O_{11}$, 1121.7087; found, 1121.7089.

HPLC chromatograms showing the purity of synthesized peptides

GLP-SNAC, purity: 98.4%

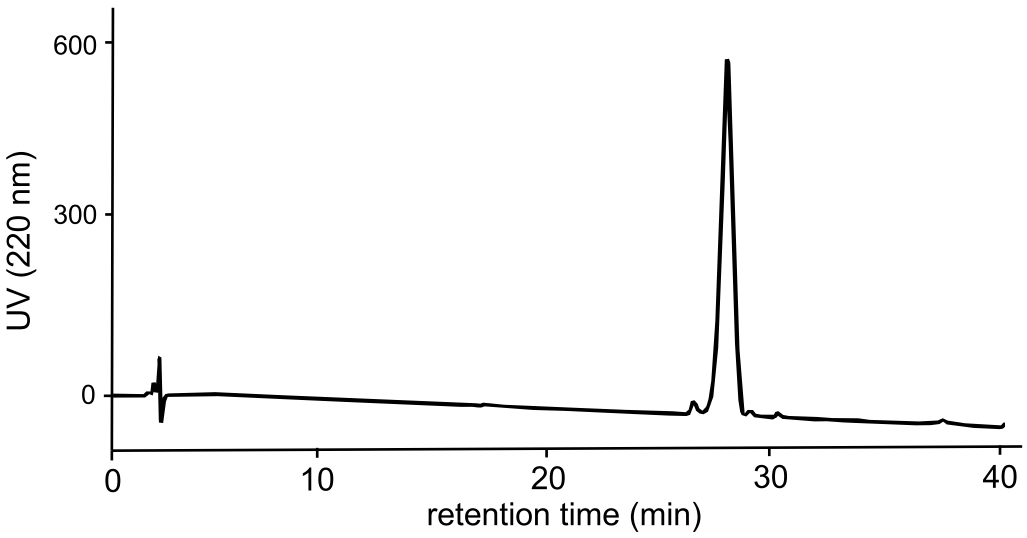

GLP-f1sA-SNAC, purity: ≥99%

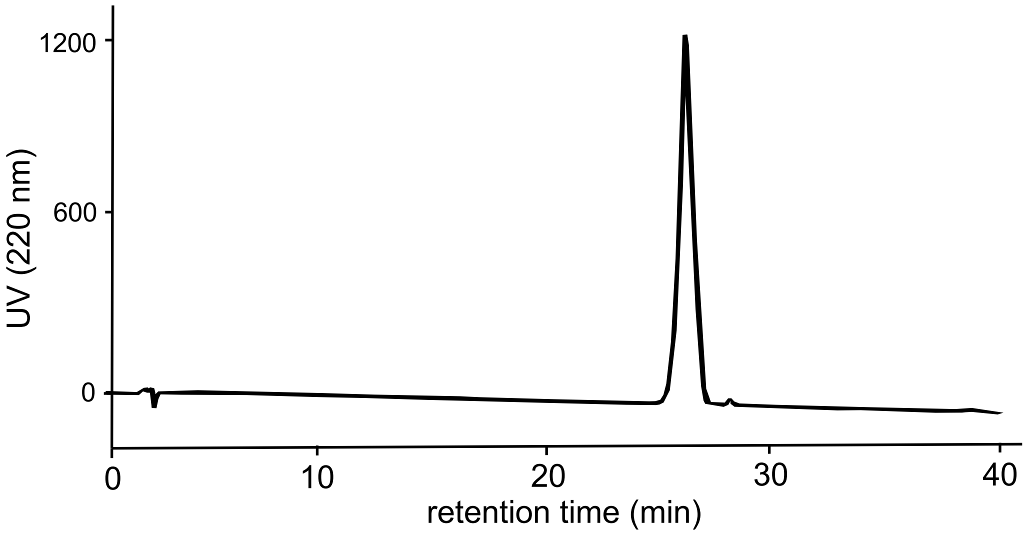

GLP-f6sA-SNAC, purity: ≥99%

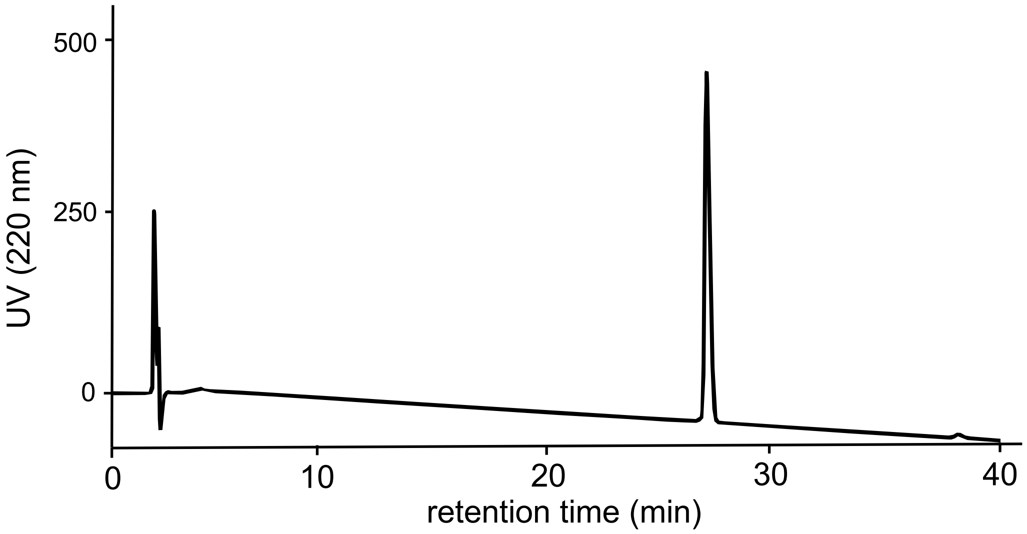

GLP-f1SA-SNAC, purity: 98.9%

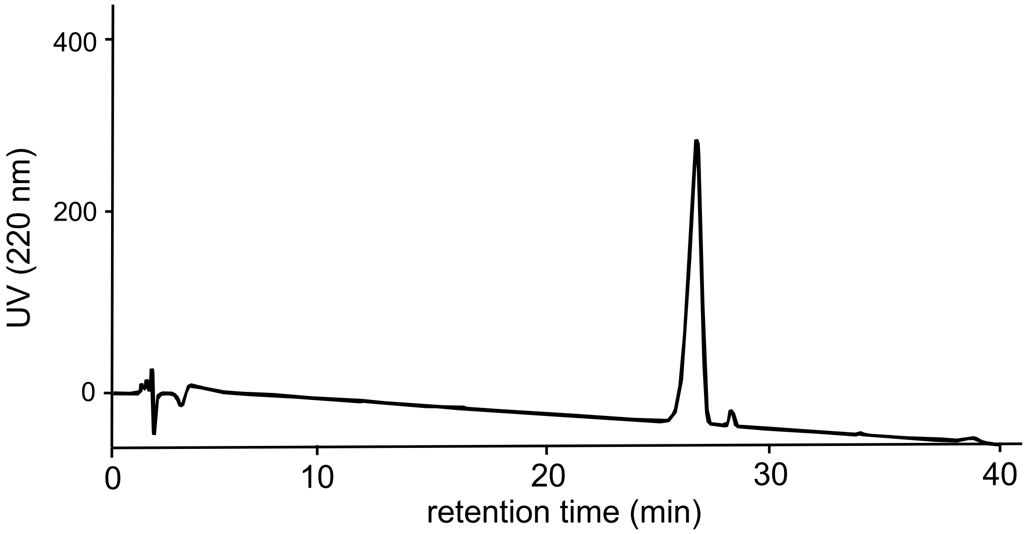

GLP-f6sA-SNAC, purity: ≥99%

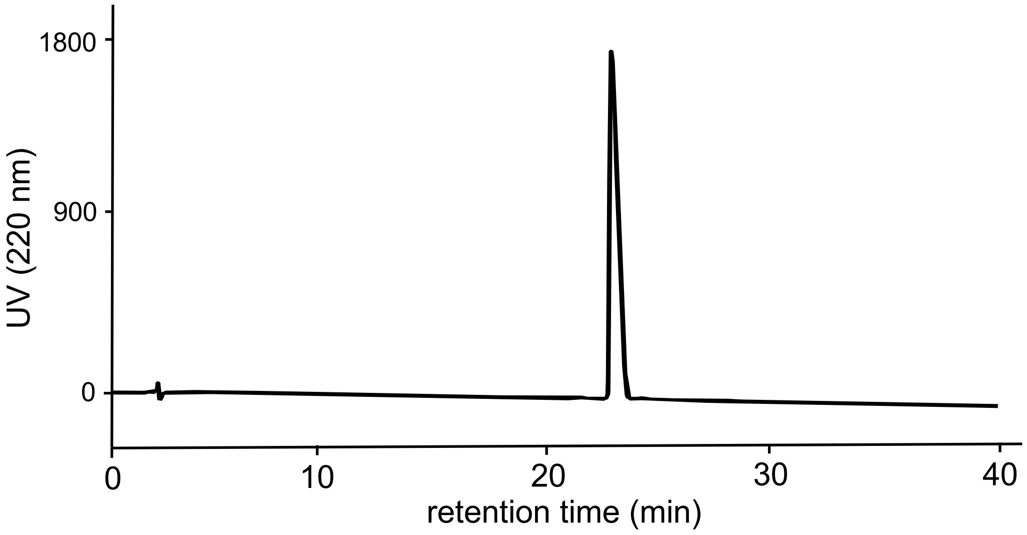

Gramicidin S (chemical synthesis), purity: ≥99%

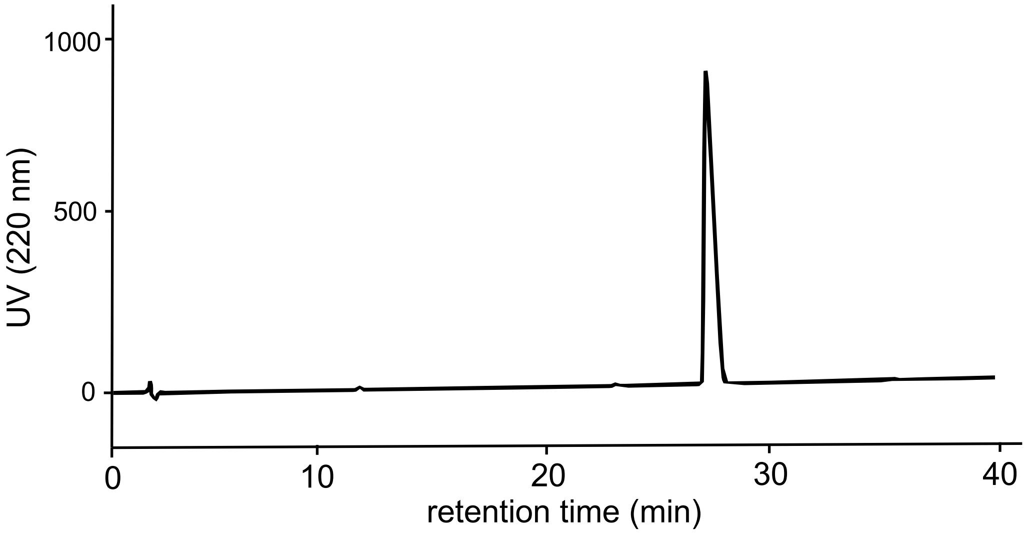

GS-SA (chemical synthesis), purity: 96.5%

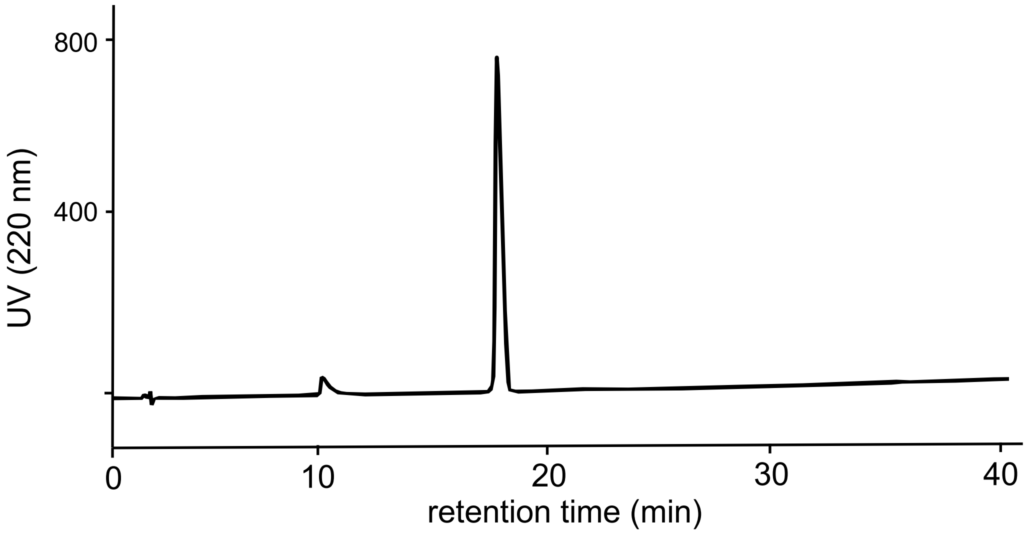

GS-sA (chemical synthesis), purity: ≥99%

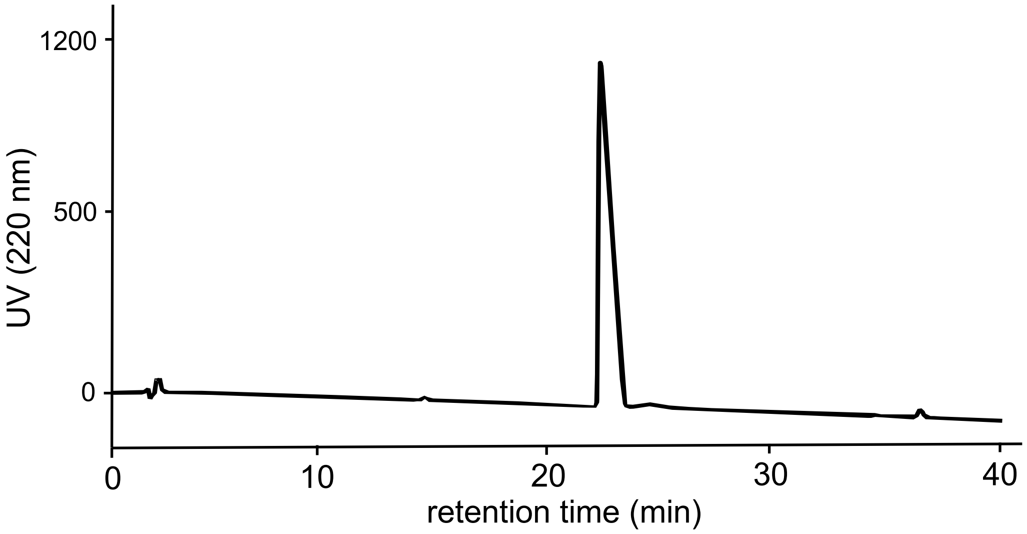

^{1}H NMR (600 MHz) and ^{13}C NMR (150 MHz) spectra of **S1** in MeOH-d_4

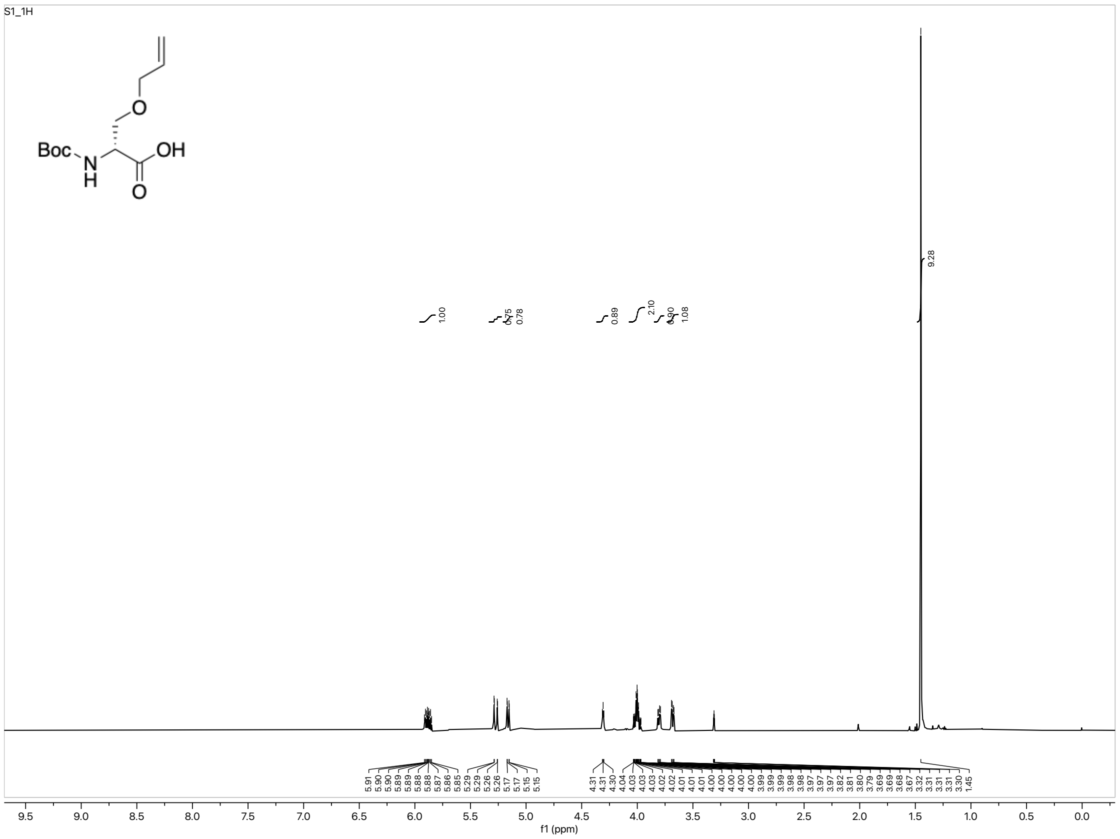

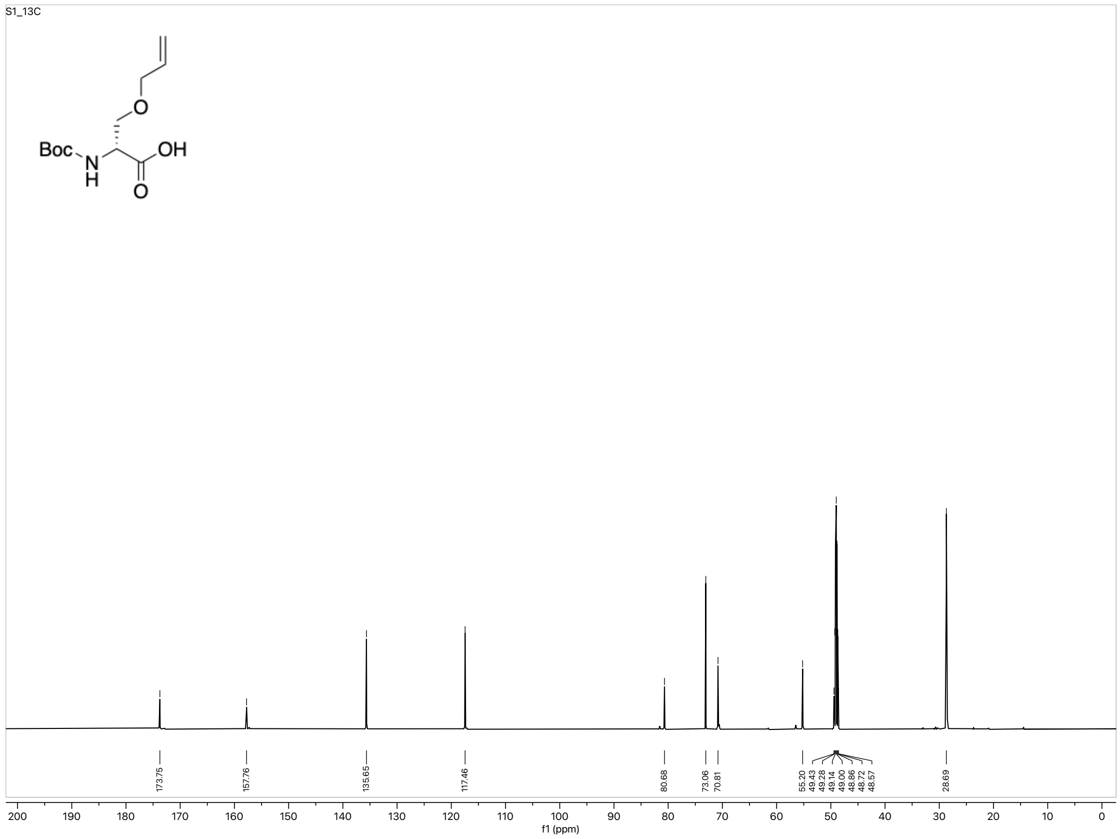

^1H NMR (400 MHz) and ^{13}C NMR (100 MHz) spectra of **S2** in DMSO-d_6

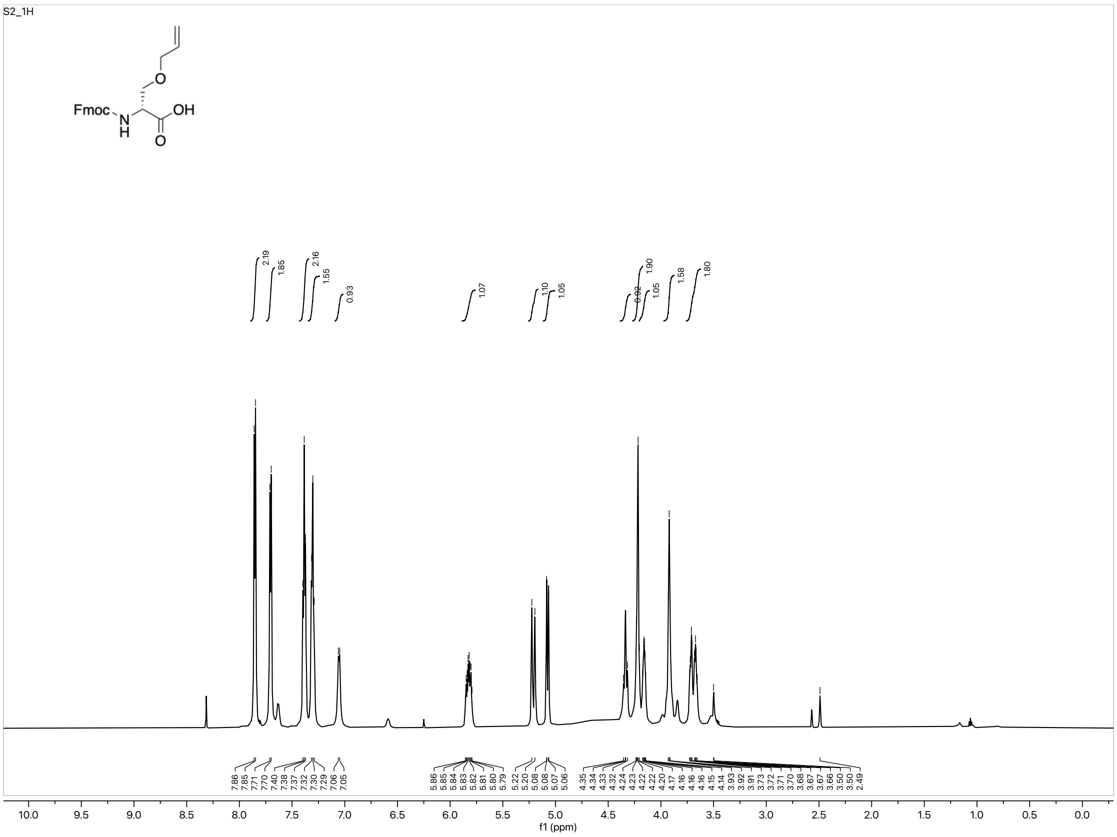

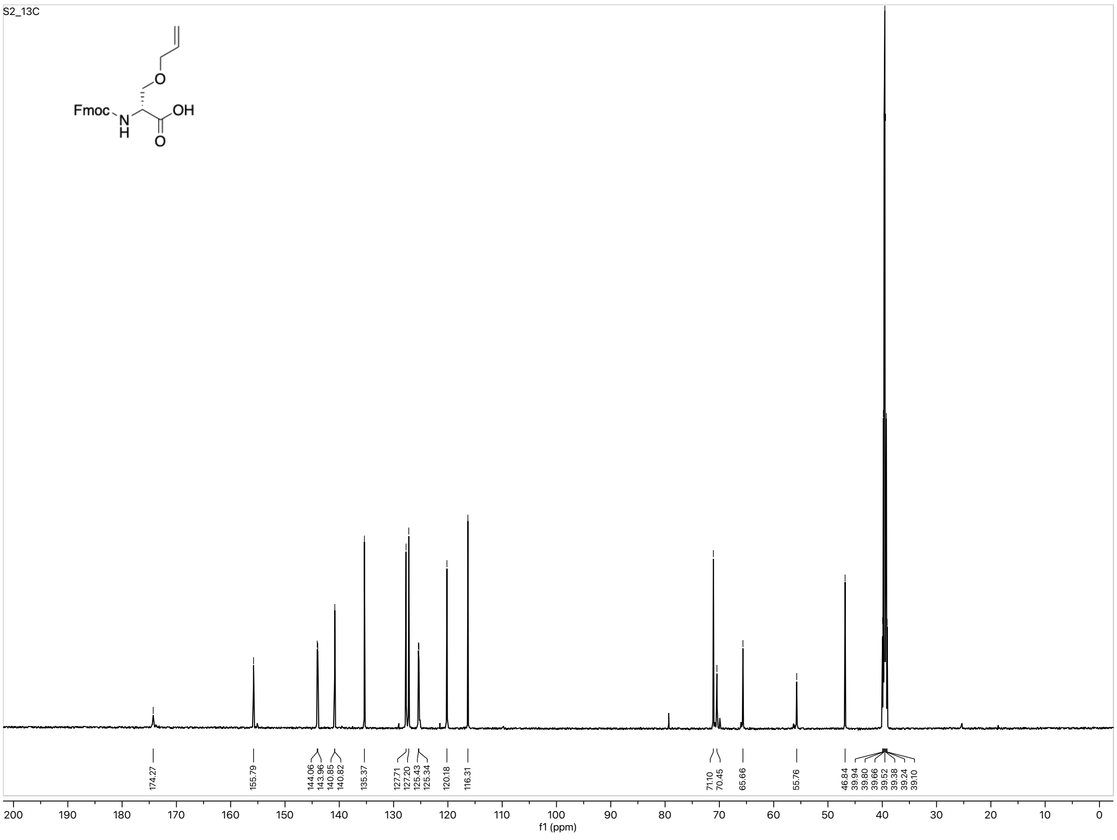

^{1}H NMR (400 MHz) and ^{13}C NMR (100 MHz) spectra of **S3** in $CDCl_3$

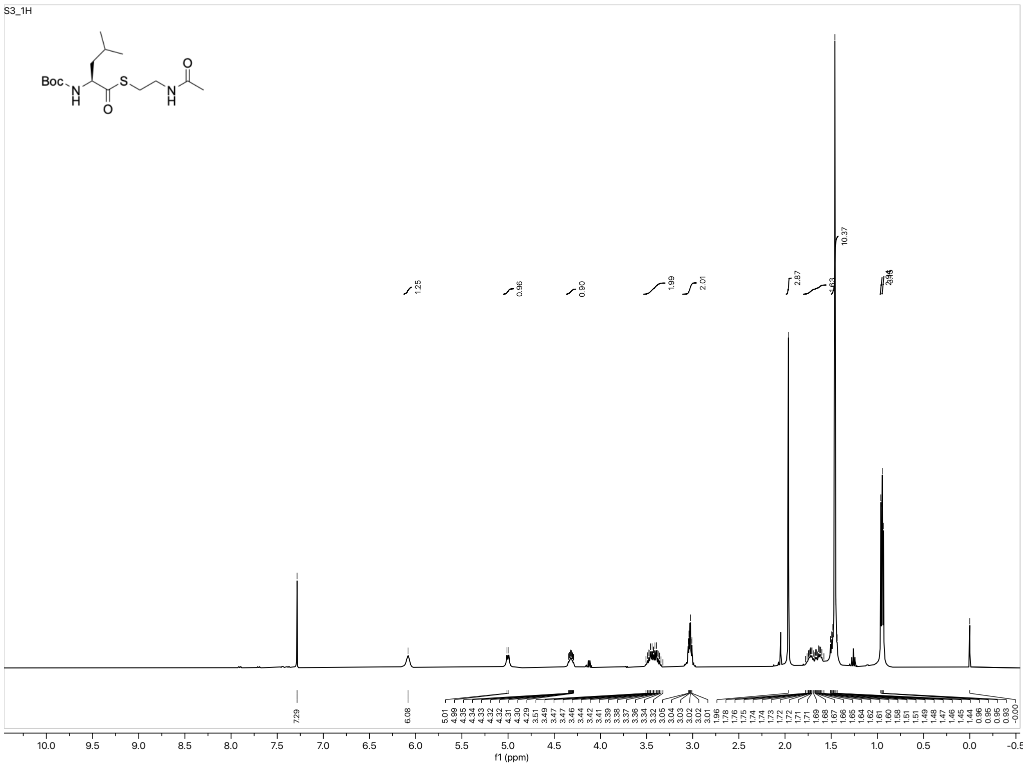

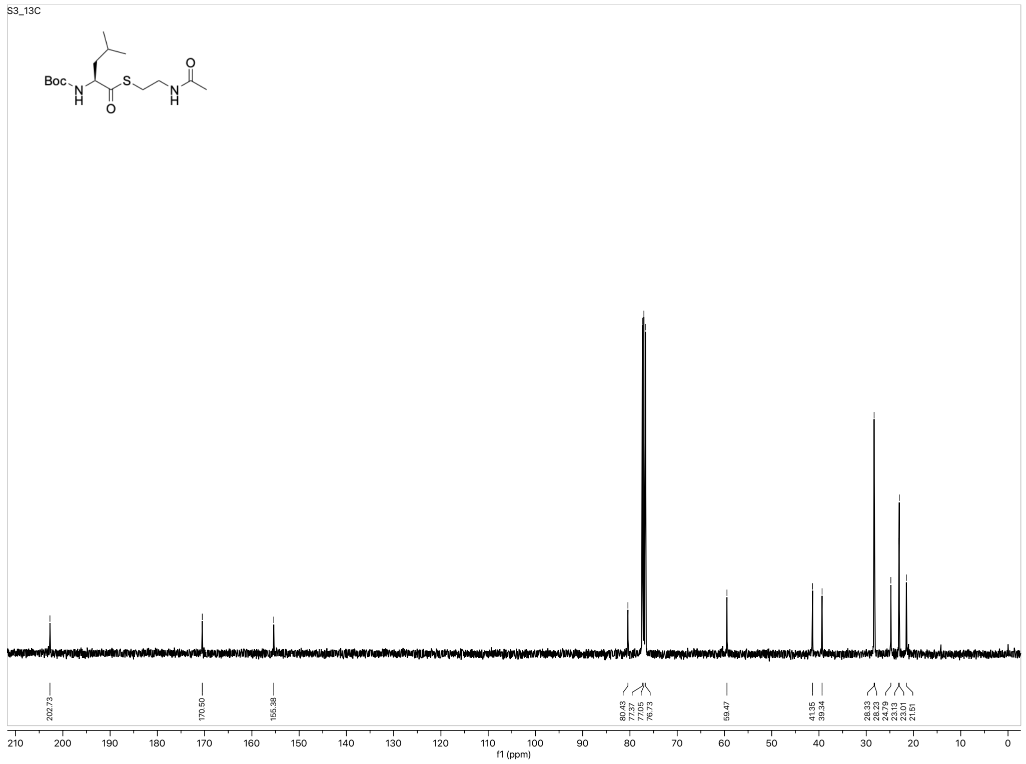

^{1}H NMR (600 MHz) spectrum of **gramicidin S** in MeOH-d_4

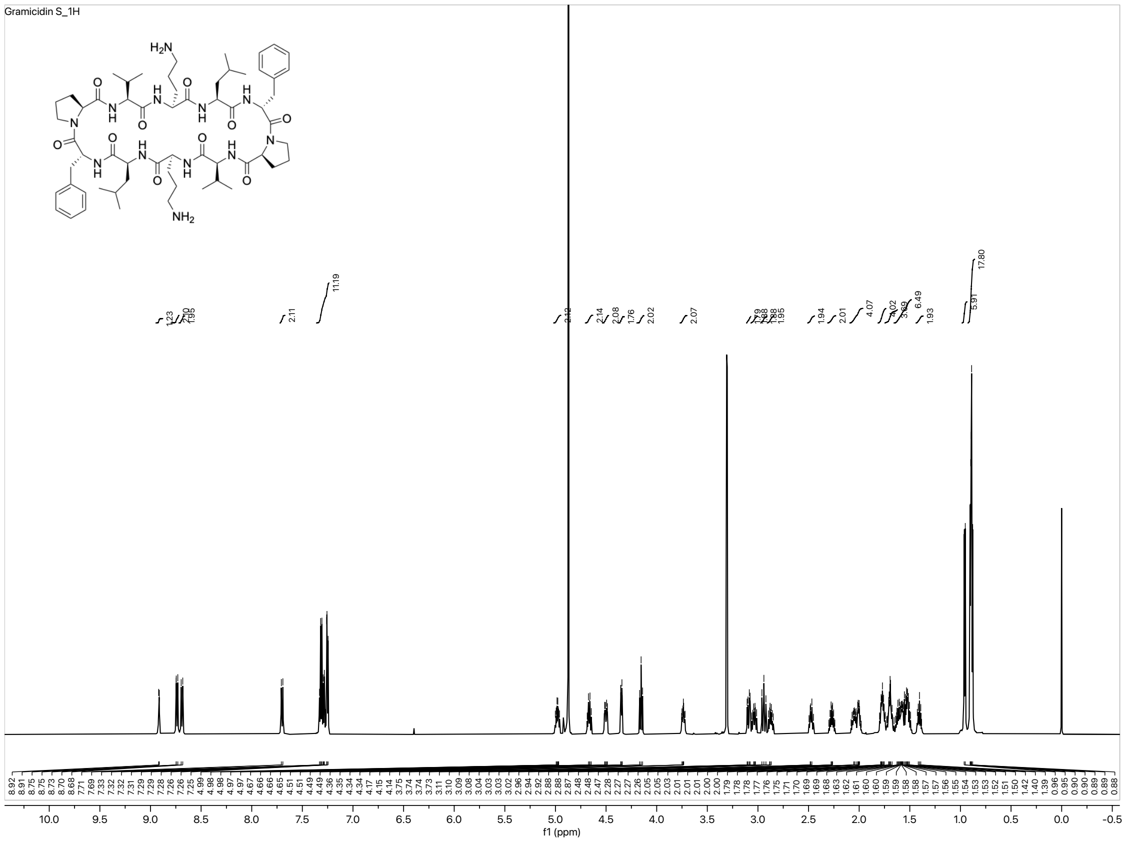

^{1}H NMR (600 MHz) spectrum of **GS-SA** in MeOH-d_4

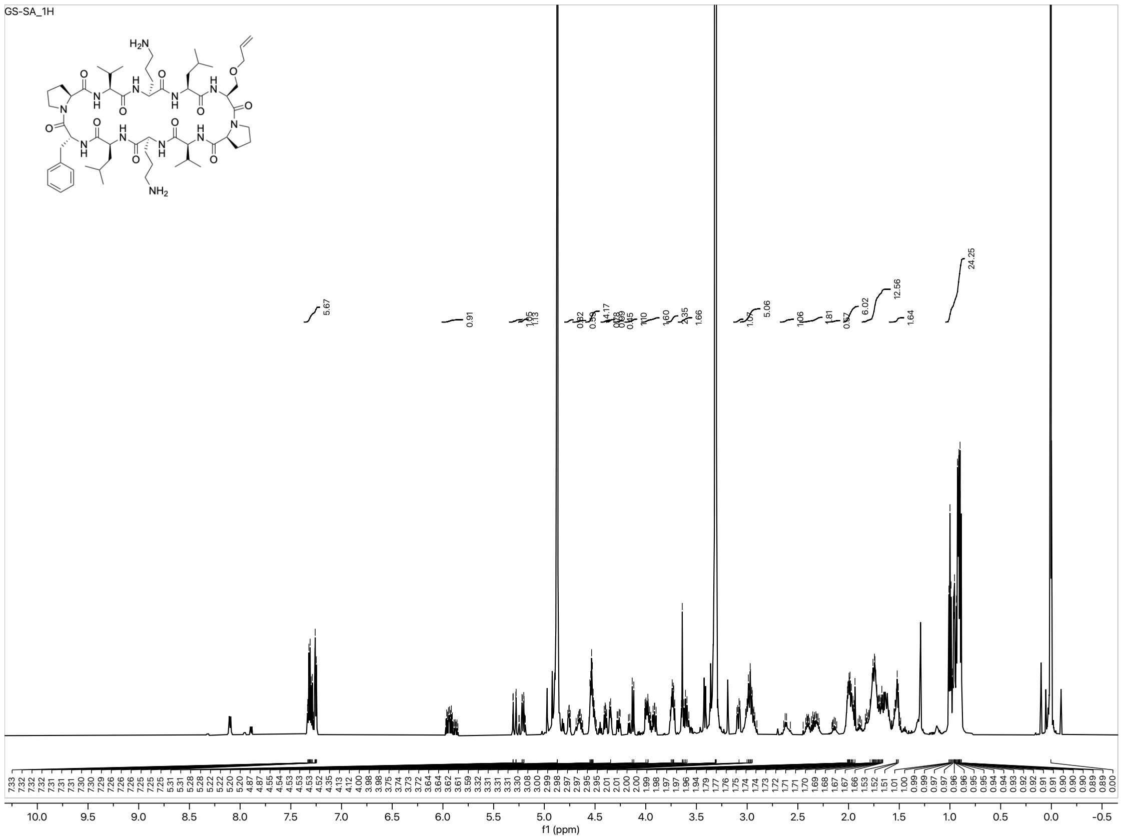

^{1}H NMR (600 MHz) spectrum of **GS-sA** in MeOH-d_4

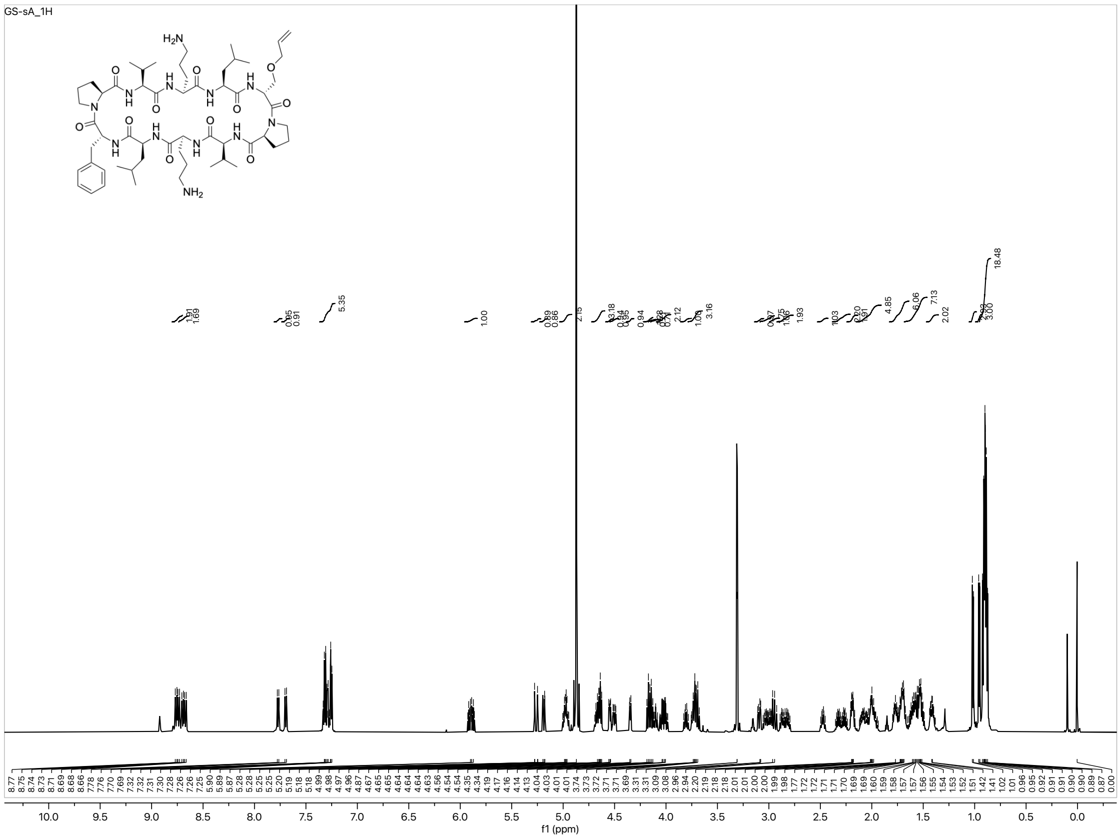

Supplement: Supplementary file 1 — Supplementary Material [file CBIC-26-e202500412-s001.pdf]
